# The use of multiple liquid chromatography methods augmented by phosphorus-31 nuclear magnetic resonance to characterize the diastereomer composition in synthetic oligonucleotides

**DOI:** 10.1016/j.chroma.2025.466600

**Published:** 2025-12-02

**Authors:** Mohsin Ali, Akanksha Manghrani, Mirandia Szramowski, Ahmed M. Abdel-Megied, Likan Liang, Nils F. Aberg, Kui Yang, Kang Chen, Yan Wang, Deyi Zhang, Steven Fletcher, Robert G. Brinson, Jace W. Jones

**Affiliations:** a Department of Pharmaceutical Sciences, University of Maryland, School of Pharmacy, Baltimore, MD, 21201, USA; b Faculty of Pharmaceutical Sciences, GC University Faisalabad, Punjab, Pakistan; c Institute for Bioscience and Biotechnology Research, National Institute of Standards and Technology and the University of Maryland, 9600 Gudelsky Drive, Rockville, MD 20850, United States; d Department of Pharmaceutical Sciences, Notre Dame of Maryland University, School of Pharmacy, Baltimore, MD 21210, USA; e Division of Product Quality Assessment X, Office of Product Quality Assessment II, Office of Pharmaceutical Quality, Center for Drug Evaluation and Research, US Food and Drug Administration, Silver Spring, MD 20993, United States; f Division of Pharmaceutical Quality Research II, Office of Pharmaceutical Quality Research, Office of Pharmaceutical Quality, Center for Drug Evaluation and Research, US Food and Drug Administration, St Louis, MO 63110, United States; g Division of Pharmaceutical Quality Research II, Office of Pharmaceutical Quality Research, Office of Pharmaceutical Quality, Center for Drug Evaluation and Research, US Food and Drug Administration, Silver Spring, MD 20993, United States; h Division of Therapeutic Performance I, Office of Research and Standards, Office of Generic Drugs, Center for Drug Evaluation and Research, US Food and Drug Administration, Silver Spring, MD 20993, United States

**Keywords:** Oligonucleotide therapeutics, Liquid chromatography, Diastereomers, Chemical activators, ^31^P NMR

## Abstract

Synthetic oligonucleotide therapeutics represent a rapidly advancing class of drugs that target RNA to modulate gene expression. The phosphorothioate modification to the nucleic acid backbone is routinely used to increase resistance to enzymatic degradation and improve the pharmacological profile. Each phosphorothioate modification creates a stereogenic center, leading to the formation of diastereomers at every modified linkage. This results in a final drug product containing a complex and heterogenous mixture of diastereomers. With the increasing regulatory approval of synthetic oligonucleotide drug products, there is a pressing need to develop analytical methods that can characterize their diastereomer composition. We detail the use of four liquid chromatography methods complemented by ^31^P NMR to characterize the diastereomer composition of fully phosphorothioated short (2- and 5-mers) and full-length (20-mers) oligonucleotide sequences. We also assessed the effect of how specific chemical activators influenced the diastereomer composition in these synthetic sequences. Our data showed that the use of multiple liquid chromatography methods augmented by ^31^P NMR provided complementary and additional insight into the diastereomer composition of phosphorothioate-linked oligonucleotides. Further, our data also indicated that the chemical activator substantially influenced the diastereomer content but other important factors including the overall chemical synthesis process and sequence specific nucleobases and modifications play an important role as well.

## 1. Introduction

Nucleic acids are an increasingly popular platform for the development of biotherapeutics to treat a wide variety of illnesses [1–4]. As of July 2025, there are 21 synthetic oligonucleotide (OGN) therapeutics approved by the U.S. Food and Drug Administration (FDA), which demonstrates the potential of nucleic acids as a platform for the development of safe and effective medicines [5]. The predominant classes of approved synthetic OGN therapeutics include antisense oligonucleotides (ASOs) and small interfering RNA (siRNA) [1]. ASOs are short, single stranded OGN therapeutics that modulate gene expression via binding to target RNA molecules (e.g., mRNA) [6]. siRNAs are also short OGN therapeutics yet comprise a duplex structure and silence gene expression via binding of the antisense strand (AS) of the duplex siRNA to target mRNA within the host cell [7]. In both cases, drug development requires overcoming the poor pharmacological properties of nucleic acids, including their rapid degradation by ubiquitous nucleases and poor drug delivery to target organs [8,9]. Chemical modifications to the nucleic acid backbone, sugar, and base increase nuclease resistance while enhancing their binding affinity and specificity [10]. The development and optimization of formulation conditions are also required to ensure that the drug is properly folded and stable at the high concentrations required for clinical administration [3]. During the drug manufacturing, several synthetic modifications are generated, including stereoisomers, which need to be identified and evaluated to ensure drug safety and efficacy [11–13].

One chemical modification commonly employed for OGN therapeutics involves the conversion of the natural phosphodiester (PO) nucleotide linkage into a phosphorothioate (PS) linkage [14]. This modification confers nuclease resistance and enhances protein binding [10,15]. A result of the PS linkage is a newly introduced chiral center at the phosphorous center creating 2^*N*^ diastereomers, where *N* is the number of PS linkages [16]. A 20-mer ASO (e.g., Tegsedi) potentially yields 2^19^ (524,288) stereoisomers. Characterization of PS diastereomer composition is highly consequential. PS stereoisomer mixtures alter the pharmacological effect and impact drug efficacy and dosing [17]. Additional considerations include uncharacterized and/or uncontrolled PS diastereomer compositions adversely impact active ingredient characterization and sameness assessment. These are critical for accurate drug labeling and development of generic drug options.

OGN therapeutics are manufactured using solid-phase chemical synthesis to build the desired nucleotide sequence [18]. This process is advantageous for incorporating chemical modifications, such as the PS linkage, due to the stepwise nature of the chemistry. The ratio and distribution of *R*p and *S*p diastereomers influence the therapeutic performance of OGNs [15,18,19]. Specific PS configurations dictate three-dimensional structure and hydrophobicity [20]. The *S*p diastereomer exposes its sulfur atom outward making it more hydrophobic than the *R*p form [21]. These structural changes impact the binding affinity and biological activity. The choice of activators during chemical synthesis affects the diastereomeric composition of PS-modified OGNs. In general, more acidic activators, such as ETT, 5-(benzylthio)-1H-tetrazole (BTT), and pyridinium trifluoroacetate (PTFA), favor formation of the *R*p diastereomer, while less acidic ones, like 4,5-dicyanoimidazole (DCI) and imidazolium triflate (ImTf), bias synthesis toward the *S*p diastereomer [22]. Currently, FDA-approved PS-modified OGN drugs are synthesized in a stereo-random/stereo-uncontrolled manner, producing a statistical mixture of all possible diastereomers [22]. Despite these trade-offs, PS modifications improve pharmacokinetic properties such as stability, nuclease resistance, and uptake, making them essential to OGN therapeutics and contributing to their clinical success. Characterizing PS stereoisomer composition is crucial for optimal drug efficacy, dosing, and generic drug development. Thus, there is the need to have sensitive and practical methods to assess the overall diastereomer composition of the final PS-linked drug product.

The analytical strategies to structurally assess the complex nature of PS-linked OGNs involve many techniques including liquid chromatography (LC), spectroscopy (e.g., NMR, UV, CD), and mass spectrometry (MS) including the recent use of ion mobility [12,23–30]. Specifically for the separation of diastereomers, LC has been a prominent method [21,31–33]. Early on Stec et al. analyzed various di- to hexadecamers OGNs with two PS modifications using ion-pair reversed-phase (IP-RP) LC [34,35]. Over the years IP-RP has been used extensively to investigate synthetic OGN chemical structure including diastereomer composition [21,31,36–38]. Various IP-RP chromatographic parameters including stationary phase chemistry, ion-pair reagent, solvent type and pH, and column temperature affect the diastereomeric resolution. In addition, nucleobase type and sequence, presence and type of chemical modifications, and sequence length also greatly impact the diastereomeric characterization. Kadlecova et al. have recently investigated the effect of different ion-pair systems and column temperature for IP-RP separation of the diastereomers [36,37]. Furthermore, Chen et al. have evaluated five different column chemistries and with six different buffer systems for IP-RP separations of OGN diastereomer mixtures [39]. Several other studies have investigated various IP-RP parameters and their effects on chromatographic separation of synthetic OGNs including impurities and chemical modifications [40–42]. The exponential increase in the number of diastereomers with the number of PS linkages drastically hinders the ability of IP-RP to separate individual stereoisomers. The partial separation up to sixteen diastereomers utilizing IP-RP chromatography has recently been demonstrated [21]. However, separation of sixteen diastereomers corresponded to a pentamer or longer length of homomeric sequences with four PS linkages at various positions. At present, the separation of diastereomer mixtures from fully PS-linked OGN full length products (FLPs) is not yet achievable [38].

Other LC methods including anion exchange (AEX) [31,43,44], metal ion complexation chromatography (MICC) [31,38], and hydrophilic interaction liquid chromatography (HILIC) [28,45,46] have been used to chromatographically resolve OGN diastereomer mixtures. Of note, MICC and strong anion exchange (SAX) combined in series with a C18 column (RP-SAX) have shown complementary chromatographic resolution to IP-RP [31,38]. In the present study, we evaluated the use of four LC methods to chromatographically resolve diastereomers varying in sequence length, number of PS linkages, presence of chemical modifications, and use of different chemical activators. The four LC methods included two different IP-RP methods differing in ion-pair reagent (triethylamine acetate (TEAA) or hexylamine acetate (HAA)), MICC, and RP-SAX. These methods were corroborated with one-dimensional (1D) ^31^P NMR. Determining the extent and range of PS diastereomer composition in OGN drugs is crucial for understanding the pharmacological effect that PS stereochemistry has on drug safety and efficacy. This understanding is currently incomplete, and the results presented herein provide the opportunity to further define PS diastereomer composition for advancing OGN therapeutic development.

## 2. Material and methods

### 2.1. Chemicals and oligonucleotide sequences

The 5-mer homomeric cytidine sequences were purchased from Creative Biolabs (Shirley, New York) with deoxyribose (unmodified) or the ribose ring modified with a 2′-O-methoxyethyl (2′-MOE). The 2′ unmodified and modified sequences were synthesized with phosphodiester (PO) or phosphorothioate (PS) backbone linkages. These sequences were labeled 5m-C-PO (#4), 5m-C-PS (#5), 5m-^MOE^C-PO (#6), and 5m-^MOE^C-PO (#7), respectively (Table 1). Additional sequences purchased from Creative Biolabs included inotersen sequences with either 4,5-dicyanoimidazole (DCI) or 5-(ethylthio)-1H-tetrazole (ETT) as the chemical activator (labeled Ino-DCI-CB (#13) and Ino-ETT-CB (#14), respectively, Table 1). Inotersen analogue with all PO linkages (labeled Ino-PO-IDT (#11)) and fully PS-linked (labeled Ino-PS-IDT (#12)) were purchased from Integrated DNA Technologies, Inc. (IDT, Coralville, Iowa). The chemical activator for the IDT sequences was unknown. The 2-mer and heteromeric 5-mer sequences were synthesized in-house. The 2-mer sequences consisted of 2′-MOE cytidines with PO and PS linkages. The PS linkages were chemically activated with either DCI or ETT. These samples were labeled 2m-^MOE^C-PO (#1), 2m-^MOE^C-PS-DCI (#2), and 2m-^MOE^C-PS-ETT (#3) (Table 1). The heteromeric 5-mer sequences had PO or PS linkages (either DCI or ETT), 2′-MOE modifications, and contained cytosine, thymine, and adenosine bases (#8, 9, and 10). The in-house sequences were synthesized using a H-6 DNA/RNA synthesizer (K&A Labs GMBH, Schaafheim, Germany) following standard phosphoramidite solid phase chemical synthesis. Supplies and chemicals for the OGN synthesis were purchased from Glen Research (Sterling, VA, USA). The drug product Tegsedi was purchased from AmerisourceBergen Corporation (Conshohocken, PA, USA) and used as received. Nuclease-free water (0.2 μm filtered) from ThermoFisher Scientific (Waltman, MA, USA) was used to reconstitute OGNs. Triethylamine (≥ 99.5 %), hexylamine (99 %), acetic acid (≥ 99 %), sodium hydroxide (≥ 98 %), sodium bromide (≥ 99 %), ammonium acetate (≥ 99 %), potassium acetate (≥ 99 %) and acetic acid (HPLC) were purchased from Sigma-Aldrich (St. Louis, MO, USA). HPLC grade water and HPLC grade acetonitrile (ACN) were purchased from ThermoFisher Scientific (Waltman, MA, USA). Hydrochloric acid (ACS Reagent) was purchased from Honeywell Research Chemicals (Charlotte, NC, USA).

### 2.2. Liquid chromatography methods

All LC experiments were conducted using an Agilent 1290 Infinity III BioLC equipped with a diode-array detector (DAD) (Agilent Technologies, Palo Alto, CA, USA). The BioLC operated using OpenLab CDS Acquisition v2.7 (Agilent Technologies). The UV detection was set to monitor a wavelength at 260 nm for all LC methods.

#### Ion-pair reversed-phase (IP-RP).

The IP-RP methods consisted of using either TEAA or HAA as the ion-pair reagent. Both methods used an InfinityLab Poroshell 120 EC–_C18_ column (2.1 × 50 mm, 1.9 μm, Agilent Technologies) and maintained at 10 °C. The TEAA method consisted of solvent A and B with 15 mM TEAA in 100 % HPLC water or 100 % HPLC acetonitrile, respectively. The gradient started at 5 % B for 2 min, ramped to 20 % B over 20 min, ramped 85 % B over 2 min, returned to 5 % B in 0.5 min, and maintained at 5 % B for 8.5 min. The HAA method consisted of solvent A and B with 5 mM HAA in 100 % HPLC water or 100 % HPLC acetonitrile, respectively. The gradient started at 5 % B for 2 min, ramped to 40 % B over 20 min, ramped 85 % B over 2 min, returned to 5 % B in 0.5 min, and maintained at 5 % B for 8.5 min. The run time was 33 min with a flow rate of 0.4 mL/min for both methods.

#### Reversed-phase strong anion exchange (RP-SAX).

The RP-SAX method consisted of solvent A with 25 mM Tris–HCl, 1 M Sodium Bromide, pH 7.4 in 20 % acetonitrile. Solvent B was 20 mM NaOH, 1 M Sodium Bromide, pH=11 (Adjusted with 1 N HCl) in 20 % ACN. The columns were placed in series and consisted of an XBridge BEH C18 XP column (2.1 × 50 mm, 2.5 μm, Waters Corporation) and a DNAPac^™^ PA200 Oligonucleotide HPLC column (2 × 250 mm, 8 μm, ThermoFisher Scientific). Both columns were maintained at 21 °C. The gradient started at 2 % B for 2 min, ramped to 30 % B over 18 min, ramped to 40 % B over 5 min, returned to 2 % B in 1 min, and maintained at 2 % B for 9 min. The run time was 35 min with a flow rate of 0.3 mL/min.

#### Metal ion complexation chromatography (MICC).

The MICC method consisted of solvent A with 10 mM Ammonium acetate + 20 ppm potassium acetate in HPLC water. Solvent B was 100 % HPLC acetonitrile. The column was an ZORBAX RR StableBond C18 (2.1 × 150 mm, 3.5 μm, Agilent Technologies) and maintained at 10 °C. The gradient started at 2 % B for 2.5 min, ramped to 10 % B over 0.1 min, ramped to 40 % B over 19.4 min, returned to 2 % B in 1 min, and maintained at 2 % B for 9 min. The run time was 30 min with a flow rate of 0.25 mL/min.

#### Sample Preparation:

All synthetic sequences were assigned with a simplified naming convention as presented in Table 1. For all tested sequences, the stock solution was prepared at a concentration of 400 μM in 500 μL of nuclease free water and stored at −20 °C. Working solutions were prepared by dilution with HPLC grade water to obtain a concentration of 10 μM. The reagents were prepared as molar concentration [21]. All buffer systems were sonicated for a minimum of 60 min for proper mixing of reagents or until verified by visual inspection.

### 2.3. ^31^P NMR

#### Sample preparation:

The 2-mer and 5-mer samples were lyophilized and resuspended in 10 mM Tris- d11 buffer, pH 7.85 to a final volume of 301.6 μL. Due to their molecular weight, they were not buffer exchanged. The concentration of the samples was calculated using the absorbance at 260 nm measured on Nanodrop and extinction coefficient calculated using IDT Oligo Calculator (https://www.idtdna.com/calc/ analyzer). The pH of the 2-mer and 5-mer samples was then measured using a Hamilton SpinTrode (Reference number 238,197). The samples were then supplemented with 5 % D_2_O and 150 μM sodium trimethylsilylpropanesulfonate (DSS-d6) to a final volume of ~320 μL for NMR data acquisition (Table 2).

For the 20-mers only, the samples were buffer exchanged using 4 mL 3000 MWCO Amicon centrifugal filter into 10 mM Tris-d11, pH 7.85. In a manner similar to the short mers, the samples were supplemented with 5 % D_2_O and 150 μM DSS-d6 and brought to a final volume of 320 μL.

#### 1D ^31^P NMR data acquisition and processing:

^31^P 1D NMR spectra were collected on a 600 MHz JEOL JNM-ECZ600R spectrometer equipped with a HFX ROYAL probe with proton decoupling during acquisition. Spectra were collected at 25 °C. A relaxation delay of 1 s and acquisition time of 0.3 s (32,768 points) were utilized. The ^31^P transmitter frequency was set to 57 ppm with a sweep width of 20 ppm. The data were processed using the JEOL DELTA 6.2 software, with zero filling to twice the number of points, 3 Hz line broadening and 3rd order polynomial baseline correction. *R*p PS area was integrated from 59.6 ppm to 56.7 ppm whereas *S*p PS area was integrated from 54.0 ppm to 56.7 ppm. *R*p abundance was defined as the proportion of *R*p PS area relative to the total PS area:

$$R\text{p}\;\text{abundance}\left(\%\right)=\frac{R\text{p}\;\text{area}}{R\text{p}+S\text{p}\;\text{area}}$$

The data were exported in .asc format and plotted using an in-house python script which normalizes each spectrum to the highest peak intensity in that spectrum, effectively putting all spectra on the same scale (0–1). The PS region (54–60 ppm) was extracted and plotted. The NMR data showed the normalized intensity for each spectrum.

## 3. Results and discussion

Four LC methods were selected to evaluate the chromatographic separation of the chosen synthetic OGN sequences. These methods included two different IP-RP methods, differing by the choice of the ion-pair reagent. The ion-pair reagents represented two different hydrophobicities, with triethylamine being less hydrophobic and hexylamine more hydrophobic. OGNs are polar molecules and require a minimal amount of an organic modifier (e.g., ACN) for elution on a C18 column [21]. However, the precise elution of an OGN on a C18 column under IP-RP conditions is influenced by the type and concentration of ion-pair used, chromatographic parameters (e.g., column conditions and organic solvent), and OGN sequence (e.g., length, order, and presence of modifications) [36]. In specific consideration for the ion-pair reagent, the alkyl chain length of the amine determines whether it is considered a weak or strong ion-pair [39]. Diethylamine and triethylamine are considered weak ion pairs, while octyl and hexyl amines are considered strong ion pairs. Both buffer systems require different concentrations of the organic modifier [47]. In regards to separating OGN diastereomers, lower ion pair concentrations are typically more suitable for stronger ion pairing reagents (~5 mM of HAA) and conversely higher concentrations are used for weaker ion pairing reagents (typically 10–20 mM but concentrations up to 100 mM for TEAA have been reported) [47]. Our data indicated that concentrations of 5 mM HAA and 15 mM TEAA (with experimental parameters outline vide infra) provided optimal conditions for diastereomer separation. The choice of column, column temperature, organic solvent and gradient have been extensively covered in literature [21,36,37,39–41] and our methods started with literature precedence and then were optimized accordingly. Our data aligned well with previous literature with the choice of the column (C18 chemistry, <2 μm particle size, and short length (5 cm)), low column temperature (10 °C), and use of ACN as the organic solvent for optimized IP-RP separations of OGN diastereomers. It should be noted that optimization of these experimental parameters is laboratory dependent and should also consider laboratory instrumentation, cost and time constraints, and desired outcome.

SAX chromatography, similar to IP-RP, has a rich history in characterizing OGN diastereomers [43,48–51]. The underlying principle of SAX separations for OGNs is the differential interaction of the negatively charged PO or PS backbone with the positively charged anion exchange resin coated to the stationary phase [52]. This interaction is strongly dependent on the pH, salt content, and organic solvent across the gradient profile [53]. Recently, the in-series alignment of a short C18 column followed by a SAX column has shown to be effective in differentially retaining highly-PS linked synthetic OGNs [31]. The two columns were coupled due to their simplicity and ability to enhance chromatographic fine structure via the use of a salt, organic, and pH gradient. Similarly in our investigations, we have found the use of the in-series columns provide enhanced resolution for OGN diastereomers compared to the use of a single SAX column. Thus, we adopted, with minor modifications, the RP-SAX method from Roussis et al. [31].

The fourth LC method, MICC, has received less attention with OGN separations, yet two recent reports have highlighted unique attributes of this separation strategy that indicated its potential to chromatographically resolve OGN diastereomers [31,38]. MICC refers to the use of a metal ion to rapidly and reversibly complex with a ligand to bias chromatographic separation [54,55]. The differential strength of the metal-ligand complex determines analyte retention. The strength of the metal ion complexation is greatly influenced by the source, type, and concentration of the metal ion. Other experimental factors, including stationary-phase chemistry, solvent composition, and pH, certainly contribute to an analyte’s differential affinity for metal-ligand complexation. In the case of OGNs, where MICC has limited literature precedence [31,38], the role of these experimental parameters to explain the use of metal ion complexation to chromatographically separate OGN diastereomers is currently not well understood. It is presumed that the metal ion (e.g., K^+^ from potassium acetate) as introduced in the mobile phase will differentially interact with the negatively charged PO or PS backbone. The resulting metal ion complexation will in turn influence the OGNs chromatographic elution profile as evident by the recent work by Roussis et al. to chromatographically characterize the diastereomer composition of synthetic OGNs [31,38]. Our current work adopted the MICC method set forth by Roussis et al. [31] with minor adjustments where the use of potassium acetate was added to mobile phase A. Although another method by Roussis et al. [38] has recently highlighted the use of silver instead of potassium as the metal ion, the experimental logistics of this method (extensive pre- and re-conditioning of the column) restricted its use in our experimental approach.

### 3.1. 2-mer sequence

**Fig. 1.**
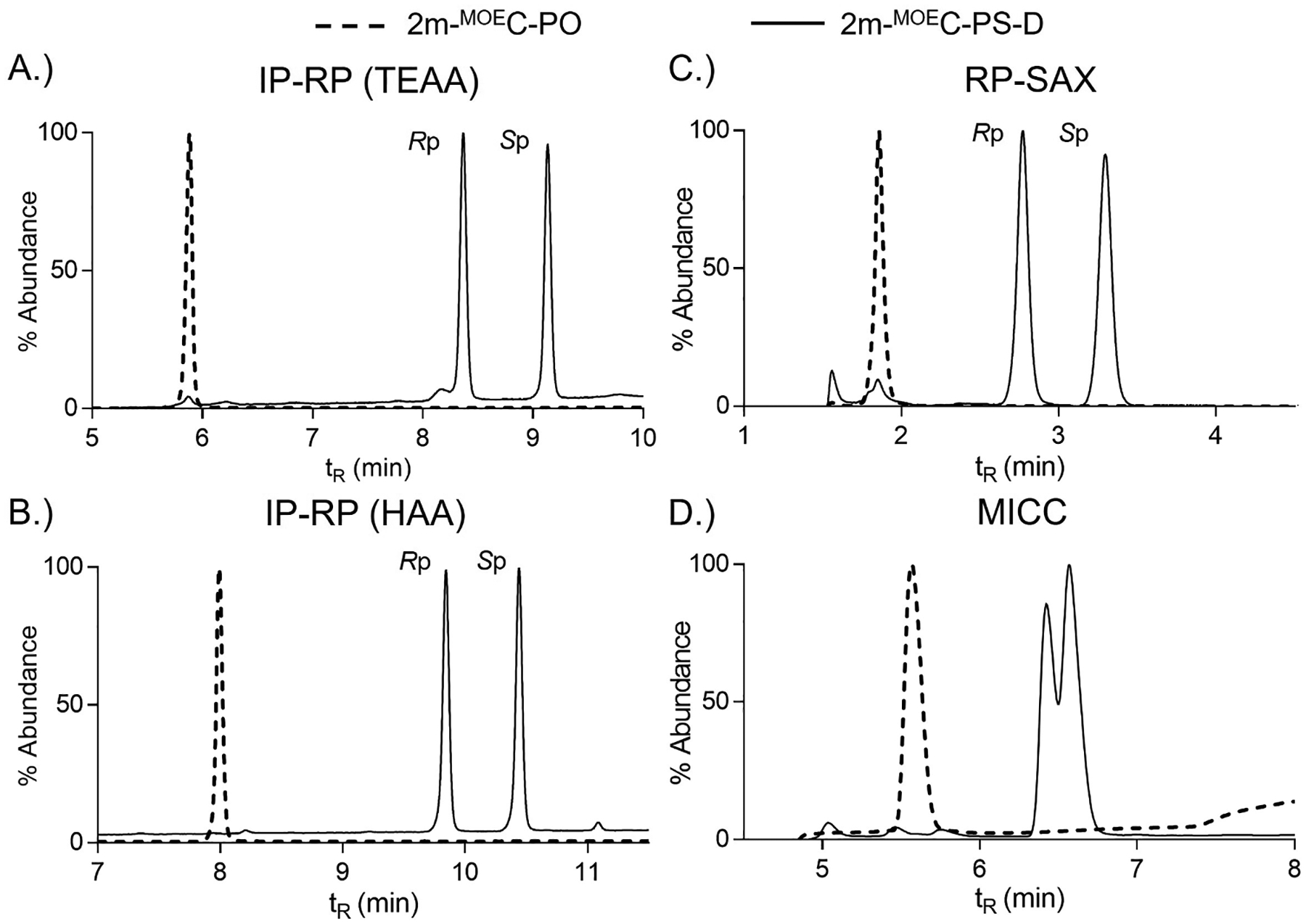
LC separation of PO- and PS-linked 2-mers. A.) IR-RP using TEAA, B.) IR-RP using HAA, C.) RP-SAX, and D.) MICC. Dashed line corresponded to 2-mer with PO-linkage, solid line corresponded to 2-mer with PS-linkage.

Our experimental approach started with a minimally complex OGN sequence which consisted of a 2-mer with 2′-MOE modified cytidines with either a PO or PS linkage (referred to as 2m-^MOE^C-PO (#1) and 2m-^MOE^C-PS-D (#2); PS-D indicates that the PS synthesis used DCI as the activator) (Table 1). The choice of 2′-MOE modified cytidine as the nucleoside centered on the relatively common use of cytosine as a nucleobase and frequent use of 2′ modifications on the sugar residue, including MOE. In addition, the OGN therapeutic used as a model sequence in these investigations was inotersen (brand name, Tegsedi) which has ten of its twenty nucleosides modified with 2′-MOE and MOE modified cytidines at the 3′ end [56]. Although the chromatographic separation of two diastereomers (one PS linkage) have been demonstrated previously [34,49,51], the separation of PO- and PS-linked 2-mers was our initial starting point to evaluate the four LC methods (Fig. 1). The 2-mer with the PO linkage (2m-^MOE^C-PO, #1) eluted out as a single, relatively sharp peak at an earlier retention time compared to the PS-linked 2-mer (2m-^MOE^C-PS-D, #2). This result was expected given the less hydrophobic nature of phosphate compared to phosphorothioate and lack of diastereomers with PO linkages and was consistent for all four LC methods. The IP-RP (both TEAA and HAA) and RP-SAX separations of the two diastereomers (*R*p and *S*p) for the 2m-^MOE^C-PS-D (#2) clearly demonstrated distinct, baseline resolved, peaks (Fig. 1A–C). The diastereomer elution order was confirmed via LC fractionation and supported by 1D ^31^P NMR spectra (supporting information (SI) Fig. S1). Consistent with literature [49], the *R*p diastereomer is less retained (elutes earlier) than the *S*p diastereomer during IP-RP and SAX separations. Of note, additional options for confirming *R*p and *S*p diastereomers, especially for more complex sequences, include the use of stereo-defined synthetic standards (when available) and use of stereoselective nuclease digestions (e.g., nuclease P1 preferentially digests *S*p diastereomers [57]).

The MICC chromatogram displayed two distinct yet not fully resolved peaks (Fig. 1D). The chromatographic resolution (R) of these two peaks was 0.64 where *R* > 1.5 is considered baseline resolved. Although further method optimization could enhance chromatographic resolution, we settled for our current MICC experimental parameters because we achieved enough resolution to clearly see two peaks, and the use of MICC with other OGN sequences revealed MICC separations may not be as suitable for small OGN sequences as other methods (e.g., IP-RP and RP-SAX). Since the stationary phase chemistry for our MICC separation was C18, it would be predicted that the elution order would follow what we observed for IP-RP and RP-SAX; *R*p elutes prior to *S*p. We observed a shift in relative abundance for the two peaks for the MICC chromatogram (Fig. 1D) compared to the other separations (Fig. 1A–C). As discussed later, our data using different activators (DCI or ETT) allowed us to comparatively assign the *R*p and *S*p diastereomers in the MICC separations, which followed our prediction of *R*p eluting before *S*p. The same samples were used for all four LC methods, and, although we expected to see the same relative abundance across the methods, the discrepancy in observed abundance could arise due to experimental variation across the methods (as observed with other sequences).

### 3.2. 5-mer homomeric sequences

Next, we moved to a longer homomeric sequence which consisted of 5-mers that were homomeric for unmodified cytidines, both with PO and PS linkages (Table 1, #s 4–7). As expected, the PO sequences eluted as a single chromatographic peak for all four LC methods (SI Fig. S2A–D). The unmodified PO sequence (5m-C-PO, #4) eluted earlier than the more hydrophobic 2′-MOE modified PO sequence (5m-^MOE^C-PO, #6) across all conditions. This was particularly pronounced in the IP-RP separation with the more hydrophobic ion-pair reagent, HAA, which had an elution peak max difference for the PO-linked sequences of 1.64 min (SI Fig. S2B). In comparison, the next closest elution peak max difference was the IP-RP with TEAA at 1.02 min (SI Fig. S2A). A similar effect was observed for the PS-linked sequences (5m-C-PS (#5) and 5m-^MOE^C-PS (#7)) where the MOE modification resulted in longer retention and thus a later elution time.

The 5-mer PS-linked sequences had 16 potential diastereomers (4 PS linkages, 2^4^=16). The rationale for choosing a 5-mer with 4 PS linkages is that the chromatographic resolution for IP-RP reaches its limit near 16 diastereomers. Further, the rationale for using a homomeric 5-mer instead of a longer sequence with 4 PS linkages (e.g., a 20-mer with 4 PS linkages) is that the length, sequence, and location of the PS linkage greatly influences the chromatographic resolution of the diastereomers [21]. Thus, the use of a homomeric 5-mer with 4 PS linkages provided controlled experimental conditions for evaluating chromatographic resolution on a model system.

TEAA represents a relatively weak (less hydrophobic) ion pair. As noted by Kadlecova et al., diastereomeric resolution is enhanced for weak ion-pair reagents [36]. Of note, we investigated the use of a weaker ion pair reagent, ammonium acetate (AmAc), and did not observe superior diastereomer resolution (data not shown). Thus, we continued our investigations using TEAA.

**Fig. 2.**
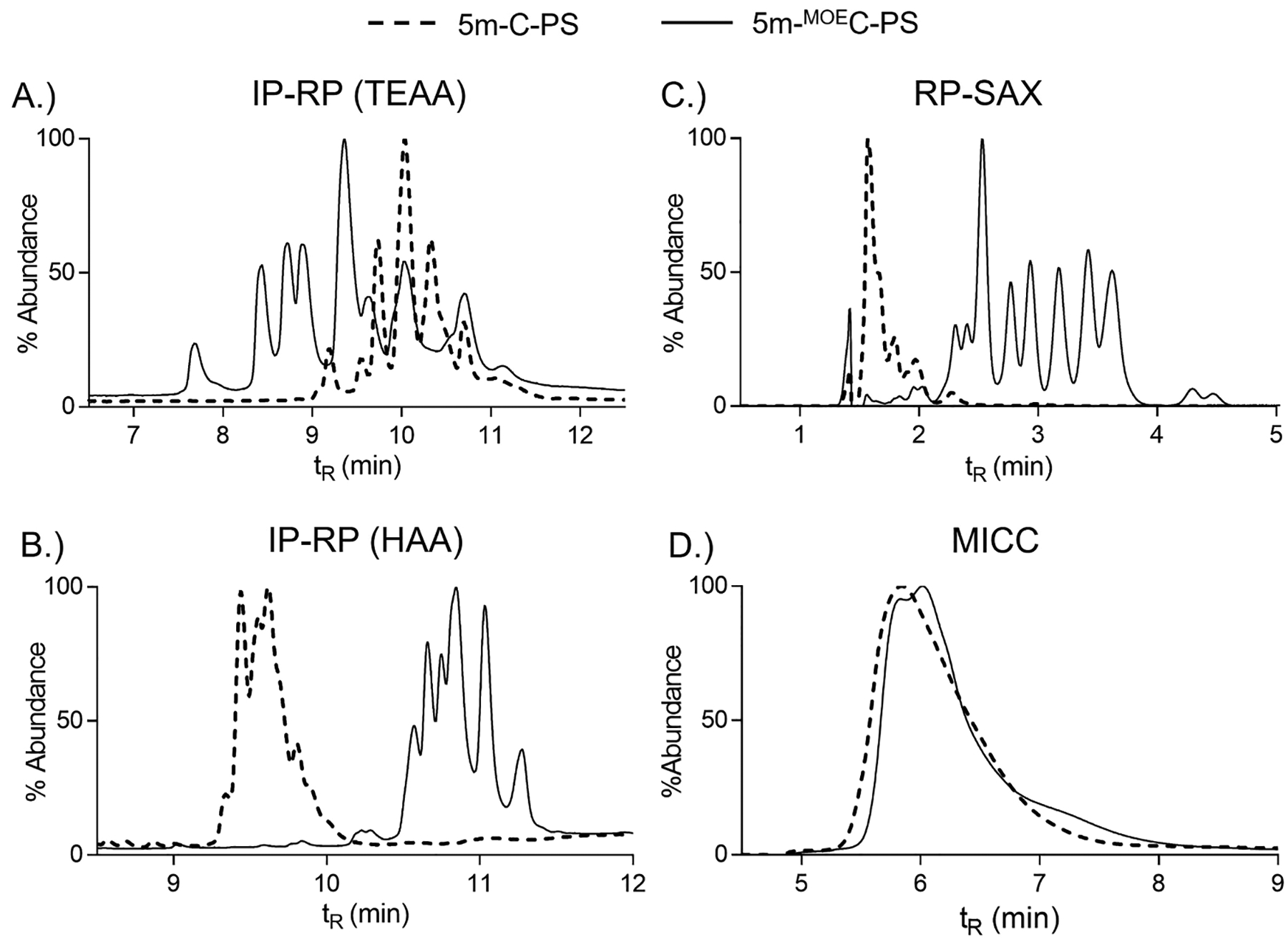
LC separation of 5-mer PS-linked homomeric cytosine with and without 2′-MOE modification. A.) IR-RP using TEAA, B.) IR-RP using HAA, C.) RP-SAX, and D.) MICC. Dashed line corresponded to 5-mer without 2′-MOE, solid line corresponded to 5-mer with 2′-MOE.

The IP-RP TEAA method was able to resolve 9 peaks (plus two shoulders) for the 2′ unmodified 5-mer cytidine PS sequence (5m-C-PS (#5), Fig. 2A, S3A). In comparison, 2′-MOE 5-mer cytidine PS sequence (5m-^MOE^C-PS, #7) showed less diastereomer resolution and displayed relatively broad peak shapes (Fig. 2A, S3B). Additional experimentation with the TEAA method by changing the column and sharpening the gradient resulted in much narrower peak shapes and increased diastereomer resolution (Fig. S4). In fact, we were able to sharpen the peak shapes and resolve, by close approximation, all 16 diastereomers in the 5m-C-PS sequence (#5) (Fig. S4A–B).

We also observed an analogous trend showing that the 2′-MOE modified sequence were less chromatographically resolved than their corresponding unmodified sequence (Fig. S4C–D). These results suggested that the relatively weak ion-pair buffer system, TEAA, was able to resolve the diastereomers of the homomeric sequence without the 2′-MOE modification as this sequence was less hydrophobic as compared to the sequence containing the more hydrophobic 2′-MOE modification. This was explained by the mix-mode nature of IP-RP separations which involve a balance between hydrophobic and electrostatic interactions with the ion pair, oligonucleotide sequence, and stationary phase [47,58]. The increased hydrophobic nature of the MOE-modified sequence had increased hydrophobic interactions directly with the C18 stationary phase providing greater retention compared to the unmodified sequence. The enhanced interaction of the MOE-modified sequence directly with the C18 column led to a comparative decrease in the electrostatic interaction with the ion-pair and the sequence. This combination resulted in overall greater retention but less diastereomer separation for the MOE-modified sequence compared to unmodified sequence.

The use of the IP-RP TEAA method with increased resolution (Fig. S4) was not pursued due to its higher variability for inter-day samples and the sharper gradient rendered these conditions ideal for the homomeric 5-mer sequences, as demonstrated here, but overall, it proved to be less robust and offered less resolution for heteromeric and longer sequences.

The HAA IP-RP method showed a contrast to the TEAA method for separation of PS-linked homomeric sequences. The HAA method displayed less chromatographic resolution compared to the TEAA method and resulted in an opposite trend for separation of the unmodified PS sequence (5m-C-PS, #5) compared to the 2′-MOE modified PS sequence (5m-^MOE^C-PS, #7) (Fig. 2B, S3C–D). These results can be explained via the use of a stronger ion-pair reagent, HAA, provided additional hydrophobic interactions between the ion-pair and the C18 stationary phase which given the current experimental conditions provided comparatively less chromatographic resolution for these homomeric sequences. In addition, the more hydrophobic ion-pair had an increased hydrophobic interaction with the 2′-MOE modified PS sequence compared to the deoxy PS sequences, resulting in a greater retention time difference between the deoxy and 2′-MOE modified sequences than was observed when TEAA was used.

In a manner similar to the IP-RP methods, the RP-SAX method displayed increased diastereomer separation for the 2′-MOE modified PS sequence over the less hydrophobic, unmodified sequences (Fig. 2C). The RP-SAX method provided relatively poor diastereomer separation for the deoxy PS sequence (5m-C-PS, #5) but did demonstrate effective diastereomer separation for the more hydrophobic 2′-MOE modified PS sequence (5m-^MOE^C-PS, #7) (Fig. 2C and S3E–F). Although this method utilized a short C18 column prior (in-series) to the SAX column, our data indicated that the observed separation was not heavily influenced by increased hydrophobic interactions in the C18 column. This observation was evident by removal of the C18 column for the SAX separation, and similar chromatograms for the four sequences were observed between the SAX and RP-SAX methods, albeit the RP-SAX separations provided increased chromatographic resolution (SAX only data not shown). The use of the short C18 column prior to the SAX column provided increased chromatographic resolution, leading to fine structure separation as reported by Roussis et al. [31]. The difference in chromatographic separation when using RP-SAX for unmodified and 2′-MOE modified PS-linked sequences was explained by enhanced electrostatic interaction of the negatively charged backbone with the cationic properties of the stationary phase when the 2′-MOE modification was present. This was unexpected given the 2′-MOE modification introduced an electronegative functional group to the sugar residue of the sequence. However, the increased chromatographic resolution, regardless of the short, in-series C18 column, indicated amplified electrostatic interaction in the SAX column. This observation suggested that the 2′-MOE modification is sterically exposing the PS backbone to more electrostatic interactions with the cationic stationary phase. In addition, these results suggested that the addition of the electronegative 2′-MOE modification to the sugar residue in the OGN sequence does directly offset the anionic state of the PS backbone. Further experiments are needed to confirm this, and we are actively investigating the use of 2′ modifications to explain the observed chromatographic behaviors.

The MICC method eluted the 5-mer diastereomers in an unresolved, broad peak (Fig. 2D). The modified sequence (5m-^MOE^C-PS, #7) was slightly more retained and presented a leading shoulder and prolonged tailing compared to the unmodified sequence (5m-C-PS, #5). Similar to the MICC 2-mer separation, additional optimization of the MICC method to achieve enhanced chromatographic resolution was carried out. However, further diastereomer separation was not achieved, suggesting that the MICC method, at least for short OGN sequences, had limited ability to resolve PS-linked diastereomers.

### 3.3. 5-mer heteromeric sequences

We next moved to short heteromeric sequences (#s 8–10). These sequences consisted of PO- and PS-linked 5-mers containing three cytidines at the 3′ end, an internal thymidine, and a 5′ external adenosine. All nucleotides contained 2′-MOE modifications. The design of these sequences followed the rationale of adding complexity by introducing different nucleobases to a 5-mer sequence and following the 3′ end sequence of our model full length product, inotersen.

**Fig. 3.**
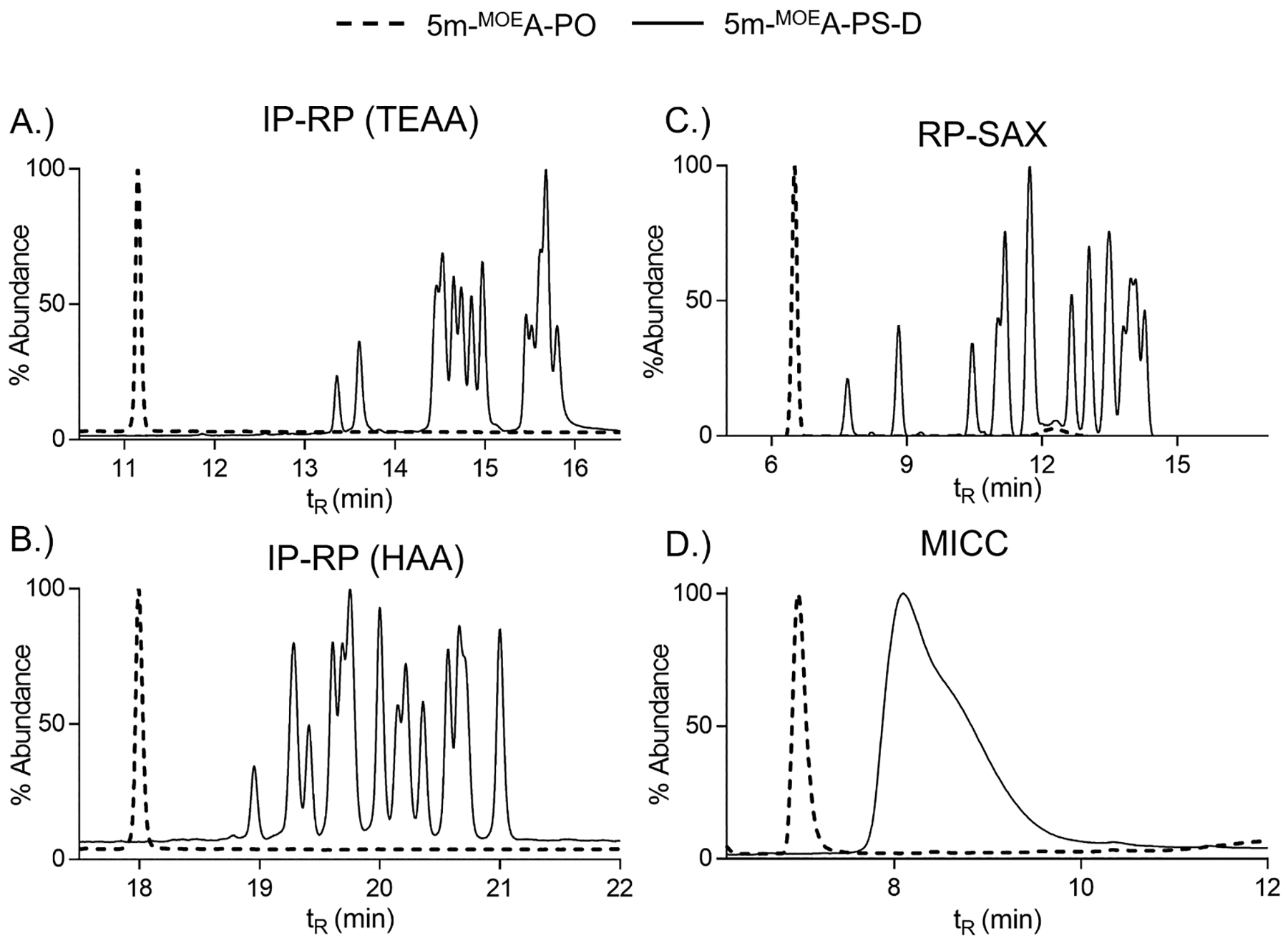
LC separation of heteromeric 5-mers with 2′-MOE modification and PO- or PS-linkages. A.) IR-RP using TEAA, B.) IR-RP using HAA, C.) RP-SAX, and D.) MICC. Dashed line corresponded to 5-mer with PO, solid line corresponded to 5-mer with PS.

As expected, the PO-linked heteromeric sequences displayed a single, sharp chromatographic peak (Fig. 3). The substitution of thymidine and adenosine to the 5′ end of the sequence in place of two cytidines substantially increased chromatographic retention for all four methods (SI Fig. S5). For example, the IP-RP TEAA method displayed a nearly 5-min increased retention for the heteromeric 5-mer compared to the homomeric cytidine sequence (SI Fig. S5A). The increased retention corresponded to the increased hydrophobicity of the thymine (T) and adenine (A) nucleobases, where nucleobase hydrophobicity follows the trend of *T* > *A* > *U* (uracil) > *C* (cytosine) > *G* (guanine) [59]. The extent of the increased chromatographic retention across all methods may not be fully explained by the increased hydrophobicity of the nucleobases. Additional electrostatic interactions, which are not fully investigated here, between the OGN and ion-pair/cationic resin/metal ion may also be contributing to the increased retention.

Like the PO sequences (discussed above), we observed, as expected, the same trend in increased chromatographic retention for the PS-linked heteromeric sequences as compared to the homomeric cytidine sequences (Fig. 3). The increased hydrophobicity of the nucleobases explained, in part, the increased chromatographic retention across all methods. We also observed increased diastereomer resolution for the heteromeric sequences compared to the homomeric sequences for the IP-RP methods (Fig. 3A–B, SI Fig. S6A–D). The increased diastereomer resolution was not only observed for the number of peaks but also peak sharpness. The weak ion-pair buffer system (TEAA) showed three well-resolved peak groupings where eleven peaks and two identifiable shoulders corresponded to the sixteen potential diastereomers (Fig. 3A, SI Fig. S6B). The stronger ion-pair buffer (HAA) displayed a more uniform spacing of chromatographically resolved peaks and had more complete resolution of the diastereomers (thirteen peaks, one shoulder) (Fig. 3B, SI Fig. S6D). IP-RP separations of OGNs are considered mix-mode chromatography, where hydrophobic and electrostatic (ionic) interactions contribute to OGN retention [47,58]. The use of a weak ion-pair (e.g., TEA) for these separations tilts the balance more favorably for direct hydrophobic interactions between the OGN and the stationary phase [60]. The electrostatic interaction between the negatively charged OGN backbone and the weak ion-pair is still present yet its overall contribution to OGN retention is reduced. Conversely, the use of a strong ion-pair (e.g., HAA) favors the electrostatic interaction of the ion-pair with the negatively charged OGN backbone and concurrently minimizing the direct hydrophobic interaction of the OGN sequence with the stationary phase [61,62]. Applying this reasoning to our IP-RP separations suggested that when electrostatic interactions are more favorable (e.g., HAA method), we observed more diastereomer separation. Yet, our experimental conditions of 15 mM TEAA and 5 mM of HAA provide only an approximate comparison given the difference in ion-pair concentrations and sliding scale of hydrophobic nature of these two ion-pair reagents. At this stage it was sufficient to say that depending on the desired outcome and chemical composition of the OGN sequence, the choice of a weak or strong ion-pair for diastereomer separation should be experimentally determined.

The RP-SAX chromatogram for the heteromeric PS-linked sequence showed similar number of peaks compared to the homomeric sequence, yet the peak shapes were decidedly sharper and considerably more retained (Figs. 3C, S6 E–F). These results followed the increased hydrophobicity of the heteromeric sequence and suggested that the more hydrophobic nucleobases (T and A) not only increased hydrophobic interactions but also influenced electrostatic interactions between the negatively charged OGN backbone and the cationic resin of the stationary phase. The MICC method for the PS-linked heteromeric sequence displayed a broad single peak with a tailing shoulder (Fig. 3D). Like the PS-linked homomeric sequence, the MICC separation did not result in diastereomer separation; both displayed a broad single peak (Figs. 2D,3D, S6 G–H). The differences in the MICC chromatograms for these two sequences were visible yet more modest compared to the other LC methods. The differences were seen in increased retention and the overall peak profile indicating the increased hydrophobicity of the OGN sequence had a differential effect on the mixed-mode nature of the MICC separation. The full effect of the retention mechanism for both RP-SAX and MICC was beyond the scope of this manuscript given the experimental conditions used were meant to be applied uniformly across varying OGNs and not specifically for mechanistic purposes. Nonetheless, our experimental data raised interesting questions about the complementary and competing hydrophobic and electrostatic interactions responsible for OGN chromatographic retention for RP-SAX and MICC separations. On-going efforts in our lab are investigating these effects.

### 3.4. Effect of chemical activator on PS diastereomer composition

**Fig. 4.**
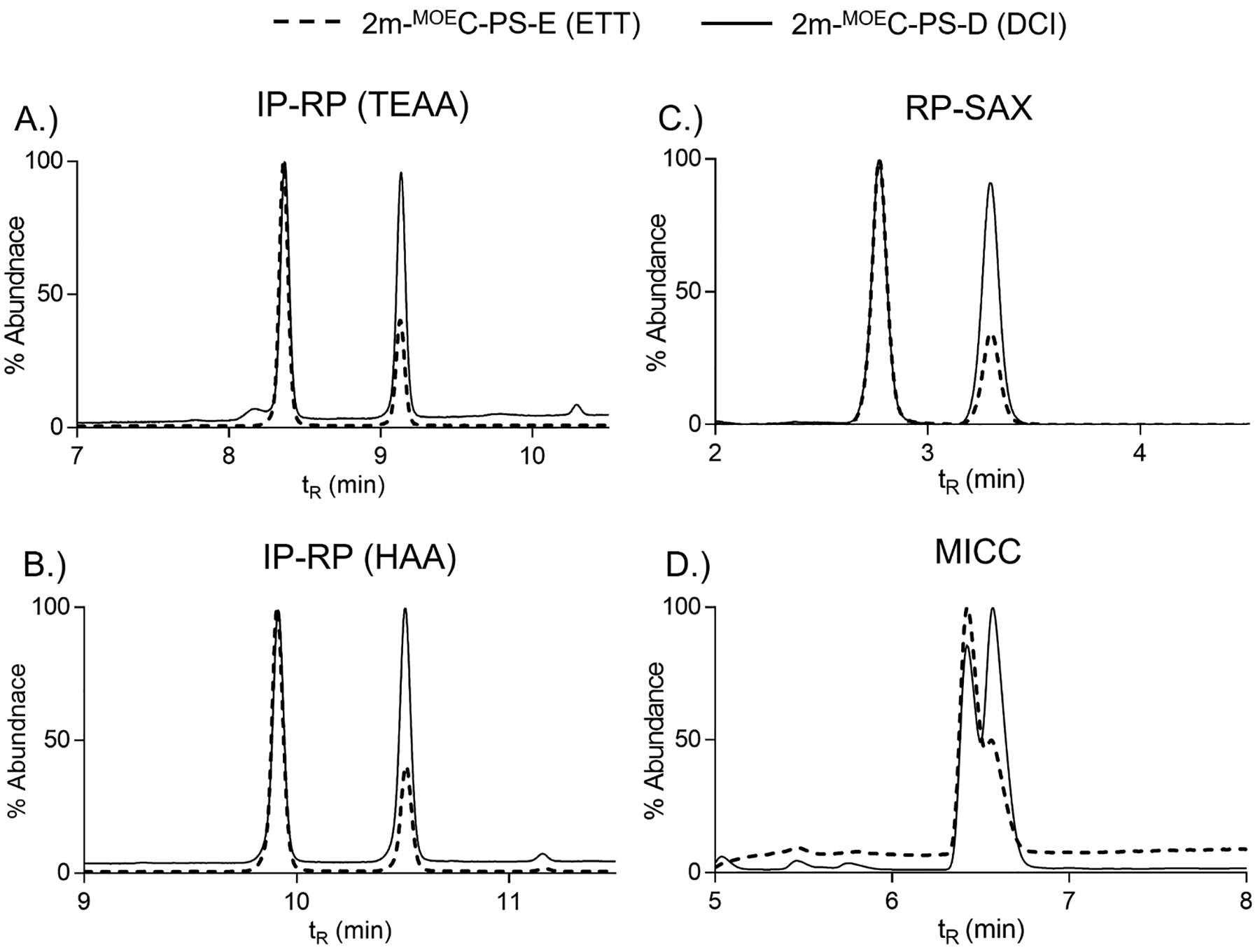
LC separation of 2-mers with PS-linkages activated with DCI or ETT. A.) IR-RP using TEAA, B.) IR-RP using HAA, C.) RP-SAX, and D.) MICC. Dashed line corresponded to 2-mer PS with ETT, solid line corresponded to 2-mer PS with DCI.

**Fig. 5.**
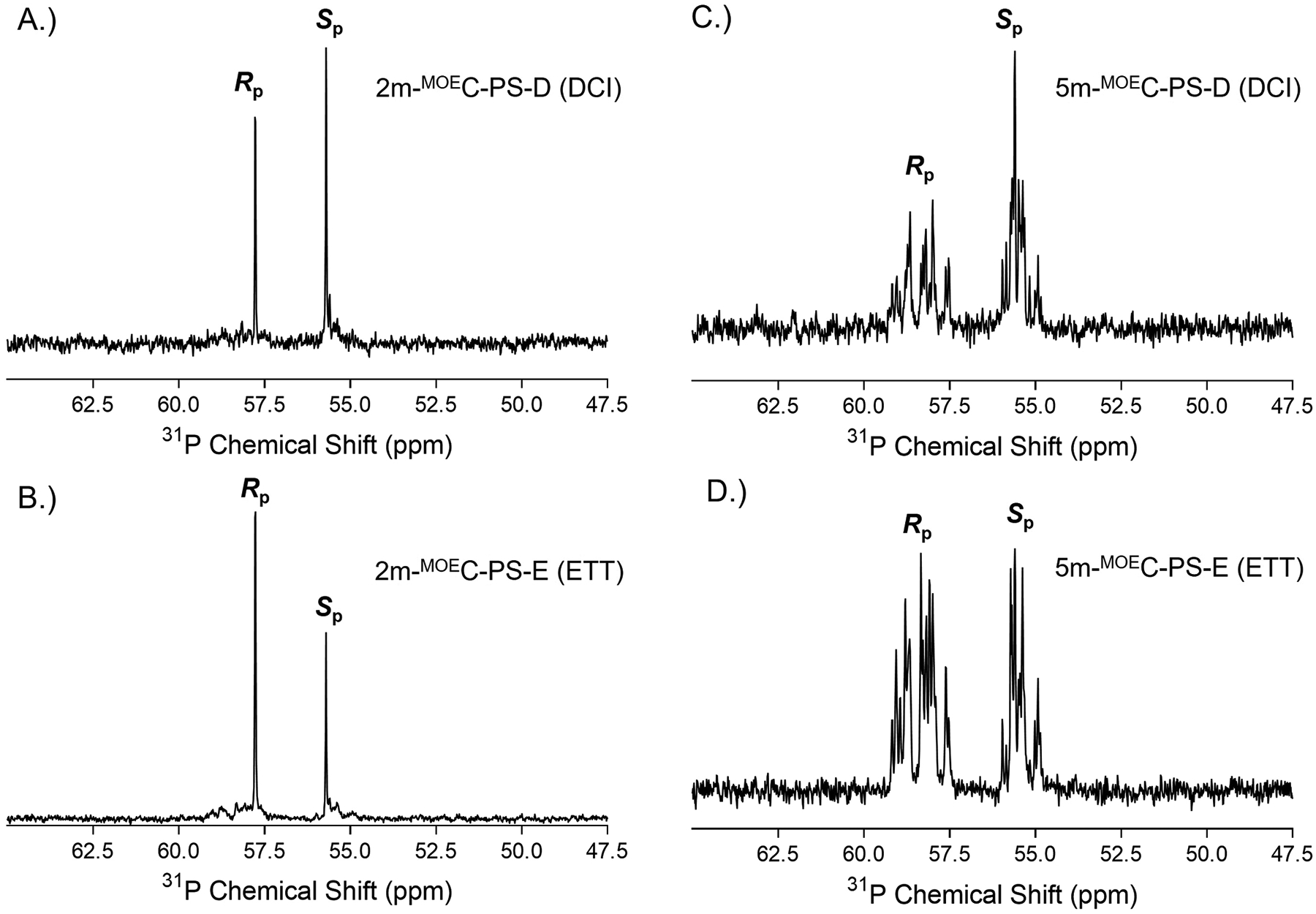
^31^P NMR spectra of PS-linked 2-mer activated with DCI (A.), 2-mer activated with ETT (B.), heteromeric 5-mer activated with DCI (C.), and heteromeric 5-mer activated with ETT (D.).

Our initial work investigated the use of two common yet chemically distinct activators, DCI and ETT, on the previously discussed 2-mer and heteromeric 5-mer sequences. The separation of the 2-mer sequences with either DCI or ETT for all four methods is shown in Fig. 4. Highly similar chromatographic profiles were seen for the two IP-RP and RP-SAX methods (Fig. 4A–C) which displayed baseline resolved *R*p/*S*p diastereomers and nearly identical ratios for *R*p/*S*p abundances. The use of DCI resulted in a near matching ratio of one-to-one *R*p to *S*p. On the other hand, the use of ETT (the more acidic activator) yielded a *R*p/*S*p ratio of approximately three-to-one. Thus, our experimental data aligns with literature where more acidic activators (e.g., ETT) produces more *R*p diastereomers [22]. Although the MICC separation did not result in baseline resolution it did follow the similar trend of DCI approximating a one-to-one *R*p/*S*p ratio and ETT favoring *R*p over *S*p in a nearly three-to-one ratio (Fig. 4D). The 2-mer sequences were also evaluated by ^31^P NMR to assess their diastereomer content (Fig. 5A–B). The ^31^P chemical shifts for PS diastereomers are well documented with the *R*p diastereomer resonating downfield of *S*p [63,64]. The ^31^P NMR spectra for the 2-mers with DCI and ETT confirmed the use of a more acidic chemical activator (ETT) favored the formation of the *R*p diastereomer and the use of a less acidic chemical activator (DCI) resulted in a comparable balance of roughly 1:1 *R*p to *S*p. The LC and NMR data were highly complementary. The minor differences in abundance as viewed via peak height represents a qualitative assessment. The quantitative assessment of how much *R*p and *S*p are present in a particular sequence is of great interest to us and current efforts in our collaborative project are pursuing the necessary analytical and statistical methods to accurately determine quantitative values.

**Fig. 6.**
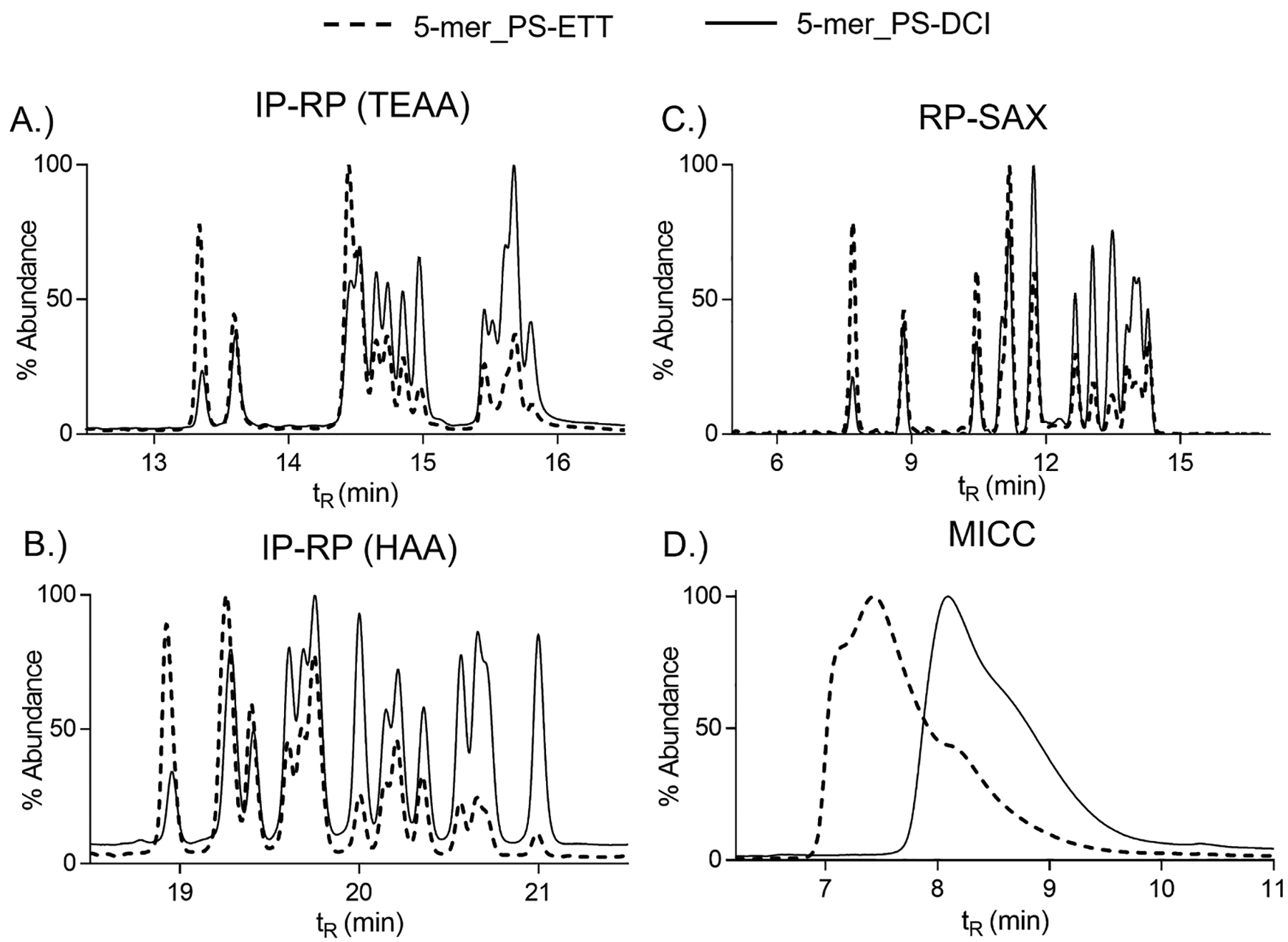
LC separation of heteromeric 5-mers with PS-linkages activated with DCI or ETT. A.) IR-RP using TEAA, B.) IR-RP using HAA, C.) RP-SAX, and D.) MICC. Dashed line corresponded to 5-mer PS with ETT, solid line corresponded to 5-mer PS with DCI.

Next, we evaluated the separation of the PS-linked heteromeric 5-mers with DCI or ETT (Fig. 6). Overall, a similar trend was observed for the 5-mers as seen with the 2-mers. The use of ETT shifted the diastereomer composition to favor the formation of the *R*p configuration (less retention, earlier peak elution), and use of DCI resulted in higher *S*p content (more retention, later peak elution). Another key aspect of the LC data was that the chromatographic profiles were identical in the number and location (t_R_ values) of chromatographic peaks, yet peak abundance was differentially shifted between the DCI and ETT sequences. This shift in abundance represented the more *S*p content for DCI and more *R*p content for ETT. Moreover, the MICC chromatogram lacked the diastereomer resolution seen in the other methods but clearly showed the shifting in retention time between the two activators which in turn visibly demonstrated the difference in hydrophobicity between more *R*p content (less hydrophobic) and more *S*p content (more hydrophobic) (Fig. 6D). The LC data was corroborated using 1D ^31^P NMR. The ^31^P NMR spectra for the PS-linked heteromeric sequences synthesized with either DCI or ETT showed well-resolved chemical shifts for *R*p and *S*p diastereomers (Fig. 5C–D). The sequence activated with DCI favored *R*p diastereomers and the sequence with ETT favored the formation of *S*p. The quantitative assessment of *R*p and *S*p content, as described for the 2-mers, is important and something we are actively pursuing.

**Fig. 7.**
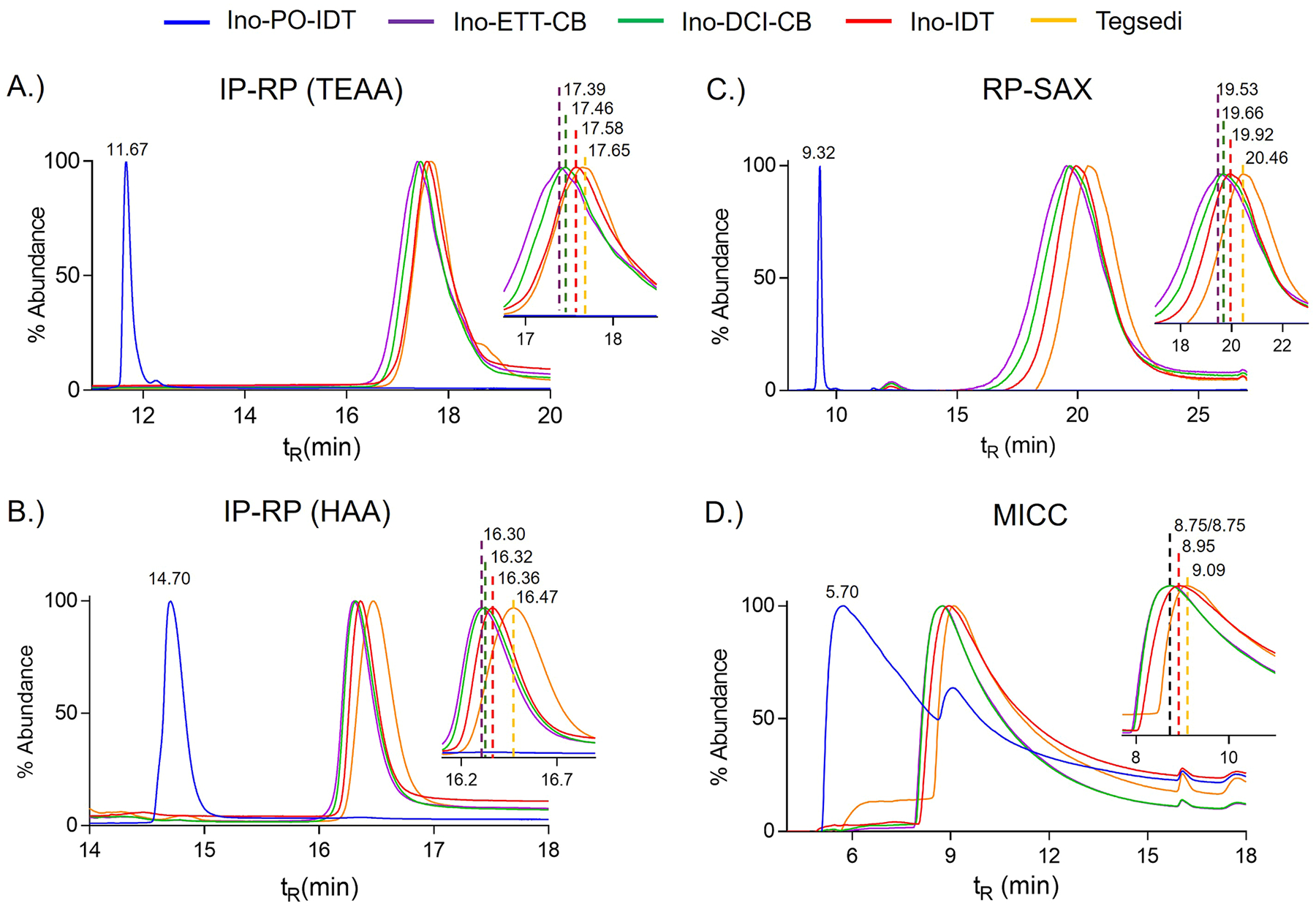
LC separation of inotersen sequences. A.) IR-RP using TEAA, B.) IR-RP using HAA, C.) RP-SAX, and D.) MICC. Blue line corresponded to Ino-PO-IDT, purple line corresponded to Ino-ETT-CB, green line corresponded to Ino-DCI-CB, red line corresponded to Ino-IDT, and yellow line corresponded to Tegsedi.

The goal of stereoisomer characterization is the translation and development of these analytical methodologies to drug product assessment of this important quality attribute. Our model drug is inotersen, including the marketed drug prodcut Tegsedi. Inotersen is a synthetic antisense 20-mer OGN [56]. The sequence of inotersen is: 5′-T^M^CTTG GTTA^M^CATGAA AT^M^C^M^C^M^C-3′ with 2′-MOE modified nucleotides at positions 1–5 and 15–20, all cytosines have a 5-methyl group (^M^C), and fully PS-linked (19 PS linkages, 524,288 diastereomer). The inotersen sequences included inotersen with unknown chemical activator (Ino-IDT, #12), inotersen synthesized with DCI (Ino-DCI-CB, #13), inotersen synthesized with ETT (Ino-ETT-CB, #14), and the FDA-approved drug product Tegsedi (#15) (Table 1). An Inotersen analogue which was fully PO-linked, referred to Ino-PO-IDT (#11) was also included in the study. The five sequences were analyzed with the four LC methods (Fig. 7). The two IP-RP and RP-SAX methods showed a well-resolved peak for Ino-PO-IDT (#11) that was chromatographically isolated to an earlier retention time compared to the inotersen sequences (Fig. 7A–C). This was expected given the PO-linked sequence contained no diastereomers and was less hydrophobic than its PS-linked counterpart. The PO-linked sequence was also visibly distinct in the MICC chromatogram displaying a broad (not well-resolved) peak that contained a gradual peak tail with a noticeable second peak (Fig. 7D). Ino-PO-IDT (#11) eluted prior to the inotersen sequences as predicted by its less hydrophobic character.

The chromatographic profiles for the inotersen sequences displayed a well-retained, broad chromatographic peak that lacked diastereomer resolution (Fig. 7). Given that inotersen contains a heterogenous and complex mixture of over 500,000 possible diastereomers this result was anticipated, yet the chromatographic retention time as measured by peak height maximum for each sequence was dependent on its chemical synthesis. We consistently observed the inotersen sequence with known activator, ETT (Ino-ETT-CB, #14), eluted first, followed by the DCI sequence (Ino-DCI-CB, #13), unknown activator (Ino-IDT, #12) and eluting last was Tegsedi (#15). We were able to achieve low variability in retention times across the four LC methods with three sigma standard deviation values of 0.03 min for the IP-RP methods, 0.05 min for RP-SAX, and 0.07 min MICC. Importantly, the high reproducibility was accomplished when samples were run within 24 h using the same solvents. The reported retention times should be viewed not as absolute values, but representative of the experimental conditions used to generate the data. That said, outside of the IP-RP HAA method comparing Ino-DCI-CB (#13) and Ino-ETT-CB (#14), the LC chromatograms yielded analytically robust data that allowed us to interpret the synthetic sequences. The first comparison where one presumable variable differed between the samples was Ino-DCI-CB (#13) and Ino-ETT-CB (#14). For these samples, the chemical synthesis was performed under identical conditions except which chemical activator was used. Our data showed the use of ETT consistently yielded an earlier retention time indicating less hydrophobic character, and conversely the use of DCI produced more retention and more hydrophobic character. These data supported our analyses of the short sequences (vide supra) and literature where the use of a more acidic activator (ETT) favors the formation *R*p and a less acidic activator (DCI) produces relatively more *S*p. The other two sequences, Ino-IDT and Tegsedi, had undisclosed chemical synthesis conditions including which activator(s) were used. Clearly both sequences had later elution times than Ino-DCI-CB (#13) and Ino-ETT-CB (#14) which indicated that these sequences were more hydrophobic and had more *S*p content than either of the known DCI or ETT sequence. The LC data showed Tegsedi, being more chromatographically retained, had the most *S*p diastereomer content.

**Fig. 8.**
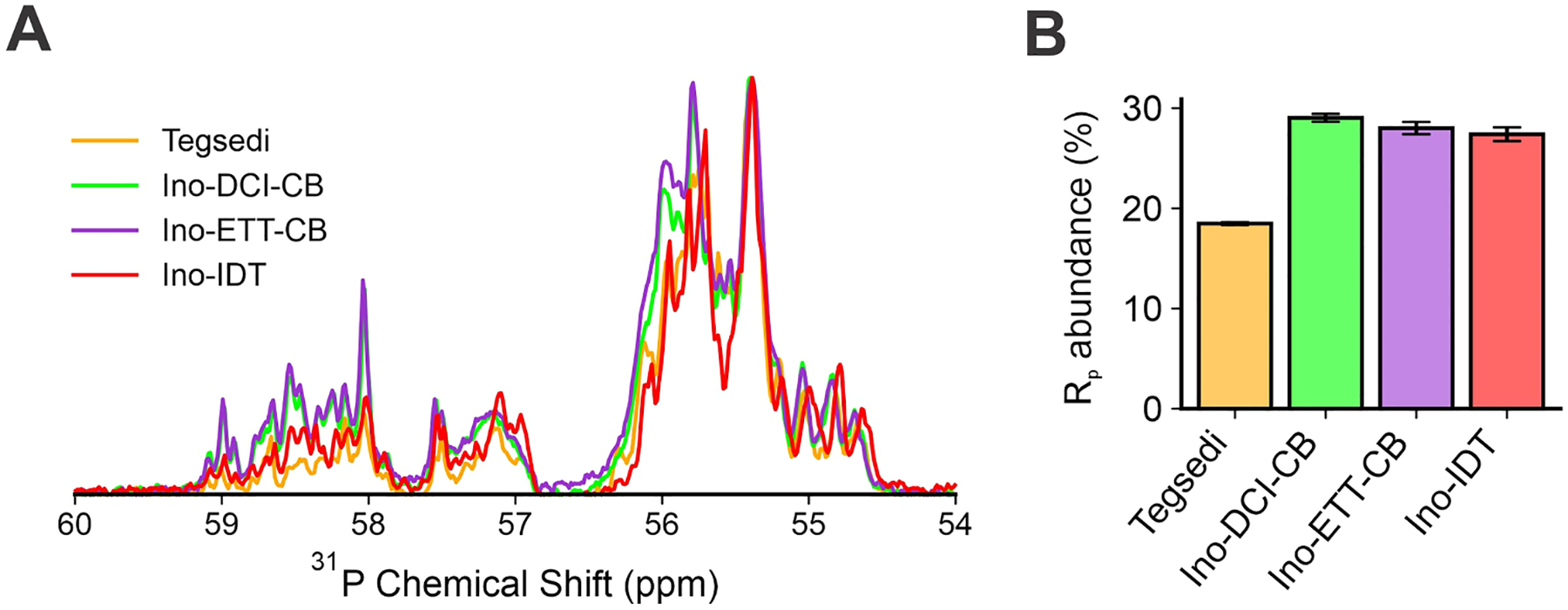
^31^P NMR spectra of Tegsedi.

The inotersen LC data was corroborated by 1D ^31^P NMR, as fingerprinting of the PS stereoisomer distribution by 1D ^31^P NMR has become one of the industry standard methods for basic PS characterization. 1D ^31^P NMR measurements revealed distinct differences between the Tegsedi and inotersen samples (Fig. 8), which were not localized on one or few discrete peaks but spread across the entire spectra. The PS region, 54 ppm to 60 ppm, is divided into two subregions. The *S*p stereoisomer is known to resonate from approximately 54 ppm to 57 ppm, and the *R*p stereoisomer resonates approximately from 57 ppm to 60 ppm. Despite the complexity of the spectra, industry standard practice is to report total *R*p and *S*p abundance from integration of each respective region, which provides a general quantitative characterization of each stereoisomer distribution. The Tegsedi *R*p abundance was approximately 18 %, whereas the other three inotersen sequences had around 28 % *R*p abundance (Fig. 8B). The result for the ino-ETT-CB construct was surprising, since ETT is known to favor *S*p stereochemistry. The manufacturer verified the use of each requested activator. It is beyond the scope of this study to investigate this observation, so an independent manufacturing source using both activators was not sought. Nonetheless, despite the surprising ETT results, 1D ^31^P NMR integration results are consistent with the chromatographic retention times across all four LC methods, with the sequence with the highest *S*p stereoisomer composition eluted last. On-going efforts in our collaborative project are underway to continue investigating the complementary use of LC and ^31^P NMR analyses to determine the diastereomer content of inotersen and other relevant OGN sequences.

Our LC and 1D ^31^P NMR data generated many intriguing questions about the role the chemical synthesis process plays in determining the diastereomer composition. The use of specific activators certainly played a substantial part in favoring diastereomer content at each elongation step. This observation was evident from our investigations into the shorter synthetic sequences up to the full-length product. The extent to which other factors (e.g., other reaction conditions, synthesis equipment, differing reagent/starting material, downstream processes etc.) influence the diastereomer composition is not well understood. Our data indicated that when the synthesis conditions were the same except for the choice of activator (Ino-DCI-CB (#13) and Ino-ETT-CB (#14)) the difference in diastereomer content was noticeable but a smaller difference was observed for the activator only-variable compared to when a different vendor was used (e.g., Ino-IDT (#12) and Tegsedi (#15)). The difference in vendor synthesis encompassed many potential variables including unknown activator(s) that were not addressable with our current experimental approach. We do not know what activator(s) were used for either Ino-IDT (#12) or Tegsedi (#15). If the chemical activator was the sole determining factor for the diastereomer ratio, our data indicated that both Ino-IDT (#12) and Tegsedi^®^ (#15) were chemically synthesized with an activator(s) other than ETT and DCI. We believe our data indicates that although the activator plays an essential role it is not the only determinate for diastereomer composition.

Another interesting question is the effect of the nucleotides involved in PS linkage. Do different nucleotide sequences result in different diastereomer ratios? What effect, if any, do nucleotide modifications have on diastereomer formation? It is assumed that the diastereomer ratio is locked into place at each elongation step. The FDA’s product-specific guidance for inotersen recommends “the R/S configuration ratio at each phosphorothioate nucleotide linkage following each elongation cycle should be measured” [65]. Thus, our data determining the diastereomer ratio of the 3′ end ^MOE^C-^MOE^C linkage with DCI and ETT should be consistent and extending this to the 5-mer should also provide a consistent diastereomer profile. What remains to be determined is the extent that the use of shorter sequences based on the full-length product sequence are indeed accurate models for diastereomer prediction.

Overall, the comparative analysis of the four LC methods revealed several interesting findings for optimizing diastereomer separation. Broadly speaking for our experimental investigation, the IP-RP and RP-SAX methods demonstrated superior diastereomer separation compared to the MICC method. Within the IP-RP methods, the choice of the ion-pair should be determined experimentally as the nucleobase type, sequence, and presence of chemical modifications greatly influenced the mix-mode chromatography. We also observed that the choice of the ion-pair buffer system had more effect on sequences with less PS-linked diastereomers. Our experimental data with inotersen demonstrated that the use of TEAA and HAA were largely similar, and the use of both ion-pair buffer systems was not necessary. Conversely, when investigating OGNs with less complex diastereomer composition (e.g., sense and antisense strands of siRNA which typically have 2–4 PS-linkages) the inclusion of multiple IP-RP methods could be advantageous for biasing diastereomer separation as seen in Fig. 3A–B where different ion-pairs yielded complementary chromatographic separation from the same sequence. The RP-SAX method, although promising and comparable IP-RP, does have some logistical disadvantages in its use of high salt and buffer concentrations. These effects can be seen in the need to be highly vigilant in the care and oversight of the LC, potential discrepancies in preparing consistent LC solvents, and limitations in interfacing to mass spectrometry. Although these issues are applicable to IP-RP methods and best laboratory practices should always be in place, the use of IP-RP methods has allowed us to efficiently navigate between complementary methods (e.g., TEAA and HAA), minimize downtime, and interface to mass spectrometry. Of note, recent advances, especially in the use of 2-dimnesional LC [66–68], offer exciting opportunities to interface LC separations (e.g., SAX) to mass spectrometry where the first-dimension solvent can be effectively “cleaned-up” in the second-dimension to allow for mass spectrometry detection. This offers an exciting approach to maximize the chromatography space without hindering the ability to gain valuable information about sequence confirmation, chemical modifications, and potential impurities.

## 4. Conclusion

The use of multiple LC methods with 1D ^31^P NMR validation provided complementary and additional insight for characterizing the diastereomer content of synthetic oligonucleotides. The use of short sequences provided unequivocal evidence on the elution order for *R*p and *S*p diastereomers and demonstrated the use of specific chemical activators impact the diastereomer ratio where a more acidic activator favors the formation of *R*p diastereomers and vice versa for a less acidic activator (relatively more *S*p). Our data also demonstrated the impact of increasing hydrophobic character of the nucleobase or sugar modifications had on the chromatographic resolution of diastereomers. An increase in sequence hydrophobicity resulted in more chromatographic resolution of the PS-linked diastereomers. The increased resolution was a combined effect of direct hydrophobic interactions and differential effects on the electrostatic (ionic) interactions per each LC method. Consequently, our data confirmed that LC experimental parameters should be optimized in a sequence specific manner to maximize the chromatographic resolution. Lastly, we demonstrated that the elution order for full-length, fully PS-linked sequences was dependent on their chemical synthesis conditions and diastereomer content. Overall, our data showed the use of specific chemical activators biased the diastereomer ratio of the synthetic OGN sequence, yet the role of other experimental parameters in the chemical synthesis and the effect of sequence-specific chemistry have not been established and therefore need further investigations.

## Supplementary Material

Supporting Information

Supplementary material associated with this article can be found, in the online version, at doi:10.1016/j.chroma.2025.466600.

## Figures and Tables

**Table 1:** List of oligonucleotides, sequence, number of PS linkages and number of diastereomers.

Number	Name	Sequence	Length	Number of PS Linkages	Number of Diastereomers
1	2m-^MOE^C-PO	^MOE^C^MOE^C	2-mer	0	None
2	2m-^MOE^C-PS-D	^MOE^C^#MOE^C	2-mer	1	2
3	2m-^MOE^C-PS-E	^MOE^C^^MOE^C	2-mer	1	2
4	5m-C-PO	CCCCC	5-mer	0	None
5	5m-C-PS	C*C*C*C*C	5-mer	4	16
6	5m-^MOE^C-PO	^MOE^C^MOE^C^MOE^C^MOE^C^MOE^C	5-mer	0	None
7	5m-^MOE^C-PS	^MOE^C*^MOE^C*^MOE^C*^MOE^C*^MOE^C	5-mer	4	16
8	5m-^MOE^A-PO	^MOE^A^MOE^T^MOE^C^MOE^C^MOE^C	5-mer	0	None
9	5m-^MOE^A-PS-D	^moE^A^#MOE^T^#MOE^C^#MOE^C^#MOE^C	5-mer	4	16
10	5m-^MOE^A-PS-E	^MOE^A^^MOE^T^^MOE^C^^MOE^C^^MOE^C	5-mer	4	16
11	Ino-PO-IDT	Inotersen (PO)	20-mer	0	None
12	Ino-IDT	Inotersen	20-mer	19	>500k
13	Ino-DCI-CB	Inotersen	20-mer	19	>500k
14	Ino-ETT-CB	Inotersen	20-mer	19	>500k
15	Tegsedi	Inotersen	20-mer	19	>500k

*C* = cytidine, *A* = adenosine, *T* = thymidine, MOE = methoxyethyl, PO = phosphodiester, PS = phosphorothioate, * = PS linkage, DCI (^#^) = dicyanoimidazole, ETT (^) = ethyl-thio-tetrazole.

**Table 2:** Sample concentration, extinction coefficient, pH and number of scans for ^31^P NMR samples.

Sample	ε_260_	Concentration (μM)	PH	Number of scans for ^31^P 1D NMR
2m-^MOE^C-PS-D	14,200	198.2	7.66	36,684
5m-^MOE^A-PS-D	45,300	68.5	7.73	65,176
2m-^MOE^C-PS-E	14,200	851.9	7.69	10,240
5m-^MOE^A-PS-E	45,300	384.1	7.58	8192
Ino-DCI-CB	190,600	500	7.85	4096
Ino-ETT-CB	190,600	500	7.85	4096
Ino-IDT	190,600	500	7.85	4096
Tegsedi	190,600	500	7.85	4096

## Data Availability

Data will be made available on request.
