# Supplementary material for: The use of multiple liquid chromatography methods augmented by phosphorus-31 nuclear magnetic resonance to characterize the diastereomer composition in synthetic oligonucleotides
Source: J Chromatogr A. Author manuscript; Available in PMC 2025 Dec 22. (PMC12718448; doi:10.1016/j.chroma.2025.466600)
Supplement: Supporting Information [file NIHMS2129124-supplement-Supporting_Information.docx]

**SUPPORTING INFORMATION**

**The use of multiple liquid chromatography methods augmented by ^31^P NMR to characterize the diastereomer composition in synthetic oligonucleotides**

Mohsin Ali^1,2^, Akanksha Manghrani^3^, Mirandia Szramowski^1^, Ahmed M. Abdel-Megied^1,4^, Likan Liang^5^, Nils F. Aberg^5^, Kui Yang^6^, Kang Chen^7^, Yan Wang^8^, Deyi Zhang^8^, Steven Fletcher^1^, Robert G. Brinson^3^, and Jace W. Jones^1^

^1^Department of Pharmaceutical Sciences, University of Maryland, School of Pharmacy, Baltimore, MD, 21201, USA; ^2^Faculty of Pharmaceutical Sciences, GC University Faisalabad, Punjab, Pakistan; ^3^Institute for Bioscience and Biotechnology Research, National Institute of Standards and Technology and the University of Maryland, 9600 Gudelsky Drive, Rockville, Maryland 20850, United States; ^4^Pharmaceutical Analytical Chemistry Department, Faculty of Pharmacy, Kafr El-Sheikh University, Kafr El-Sheikh City, Egypt; ^5^Division of Product Quality Assessment X, Office of Product Quality Assessment II, Office of Pharmaceutical Quality, Center for Drug Evaluation and Research, US Food and Drug Administration, Silver Spring, MD 20993, United States; ^6^Division of Pharmaceutical Quality Research II, Office of Pharmaceutical Quality Research, Office of Pharmaceutical Quality, Center for Drug Evaluation and Research, US Food and Drug Administration, St Louis, MO 63110, United States; ^7^Division of Pharmaceutical Quality Research II, Office of Pharmaceutical Quality Research, Office of Pharmaceutical Quality, Center for Drug Evaluation and Research, US Food and Drug Administration, Silver Spring, MD 20993, United States; ^8^Division of Therapeutic Performance I, Office of Research and Standards, Office of Generic Drugs, Center for Drug Evaluation and Research, US Food and Drug Administration, Silver Spring, Maryland 20993, United States.

**Corresponding Author:**

Jace W. Jones

Department of Pharmaceutical Sciences

University of Maryland School of Pharmacy

20 N. Pine Street, Room N721

Baltimore, MD 21201

410.706.7598

[jjones@rx.umaryland.edu](mailto:jjones@rx.umaryland.edu)

**SUPPORTING INFORMATION**

**SI Figure S1.** A.) IP-RP chromatogram of 2-mer. B.) 1D ^31^P NMR of isolated 1^st^ (left) peak from top chromatogram.

**SI Figure S2.** LC separation of homomeric 5-mers with and without 2'-MOE modification and with PO- or PS-linkages. A.) IR-RP using TEAA, B.) IR-RP using HAA, C.) RP-SAX, and D.) MICC. Blue line corresponded to 5m-C-PO, red line corresponded to 5m-^MOE^C-PO, green line corresponded to 5m-C-PS, and purple line corresponded to 5m-^MOE^C-PO.

**SI Figure S3**. LC separation of PS-linked homomeric 5-mers with and without 2'-MOE modification. A.) IR-RP using TEAA for 5m-C-PS, B.) IR-RP using TEAA for 5m-C- ^MOE^-PS, C.) IR-RP using HAA for 5m-C-PS, D.) IR-RP using HAA for 5m-C- ^MOE^-PS, E.) RP-SAX for 5m-C-PS, F.) RP-SAX for 5m-C- ^MOE^-PS.

**SI Figure S4.** LC separation of PS-linked homomeric 5-mers with and without 2'-MOE modification. A.) IR-RP using TEAA for 5m-C-PS on an Agilent RR column, B.) IR-RP using TEAA for 5m-C-PS on a Waters BEH C18, C.) IR-RP using TEAA for 5m-^MOE^C-PS on an Agilent RR column, D.) IR-RP using TEAA for 5m-^MOE^C-PS on a Waters BEH C18.

**SI Figure S5.** LC separation of PO-linked homomeric and heteromeric 5-mers with 2'-MOE modification. A.) IR-RP using TEAA, B.) IR-RP using HAA, C.) RP-SAX, D.) MICC.

**SI Figure S6.** LC separation of PS-linked homomeric and heteromeric 5-mers with 2'-MOE modification. A.) IR-RP using TEAA for 5m-^MOE^C-PS, B.) IR-RP using TEAA for 5m-^MOE^A-PS, C.) IR-RP using HAA for 5m-^MOE^C-PS, D.) IR-RP using HAA for 5m-^MOE^A-PS, E.) RP-SAX for 5m-^MOE^C-PS, F.) RP-SAX for 5m-^MOE^A-PS, G.) MICC for 5m-^MOE^C-PS, H.) MICC for 5m-^MOE^A-PS.


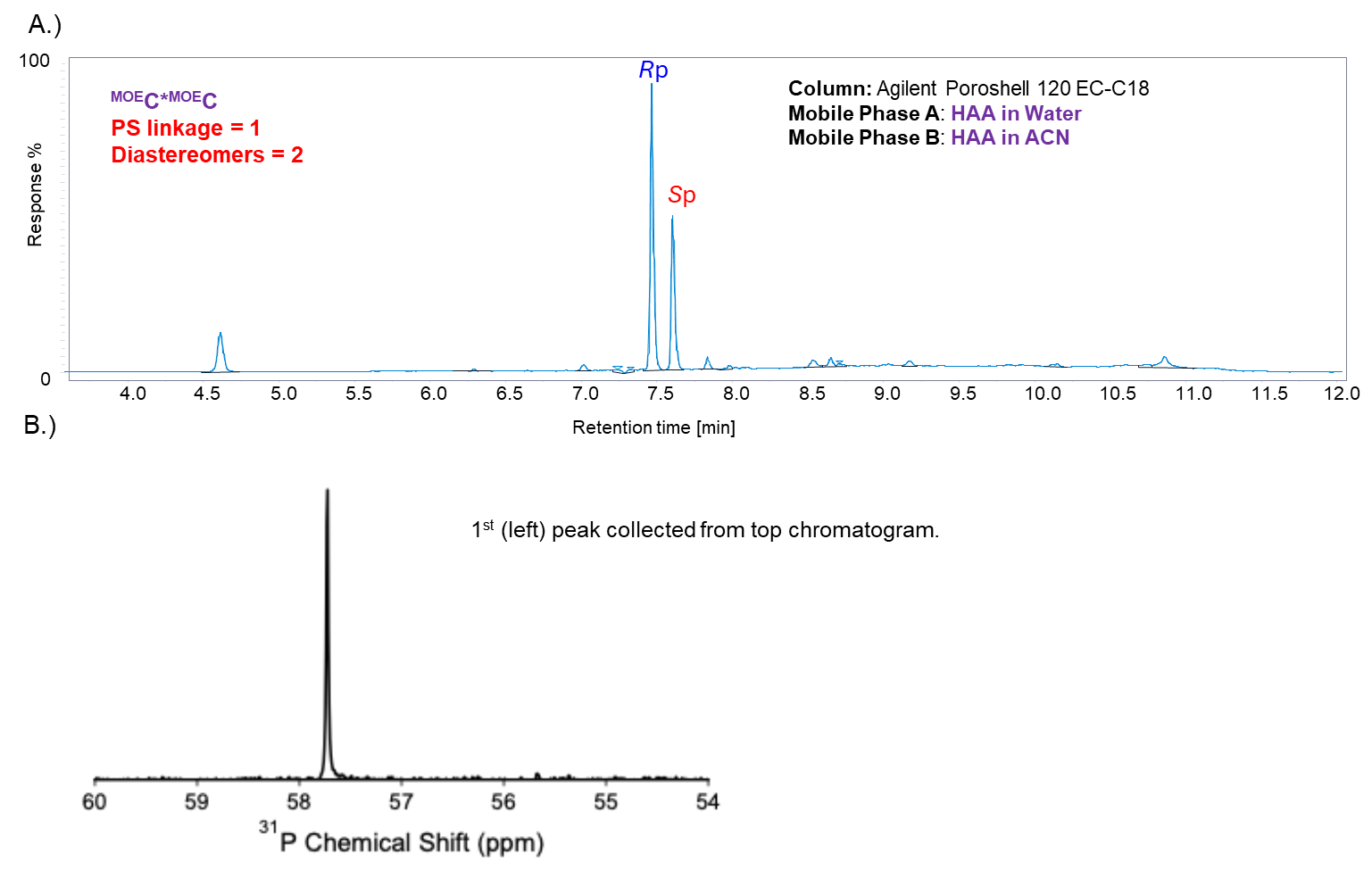


**SI Figure S1**. A.) IP-RP chromatogram of 2-mer. B.) 1D ^31^P NMR of isolated 1^st^ (left) peak from top chromatogram.


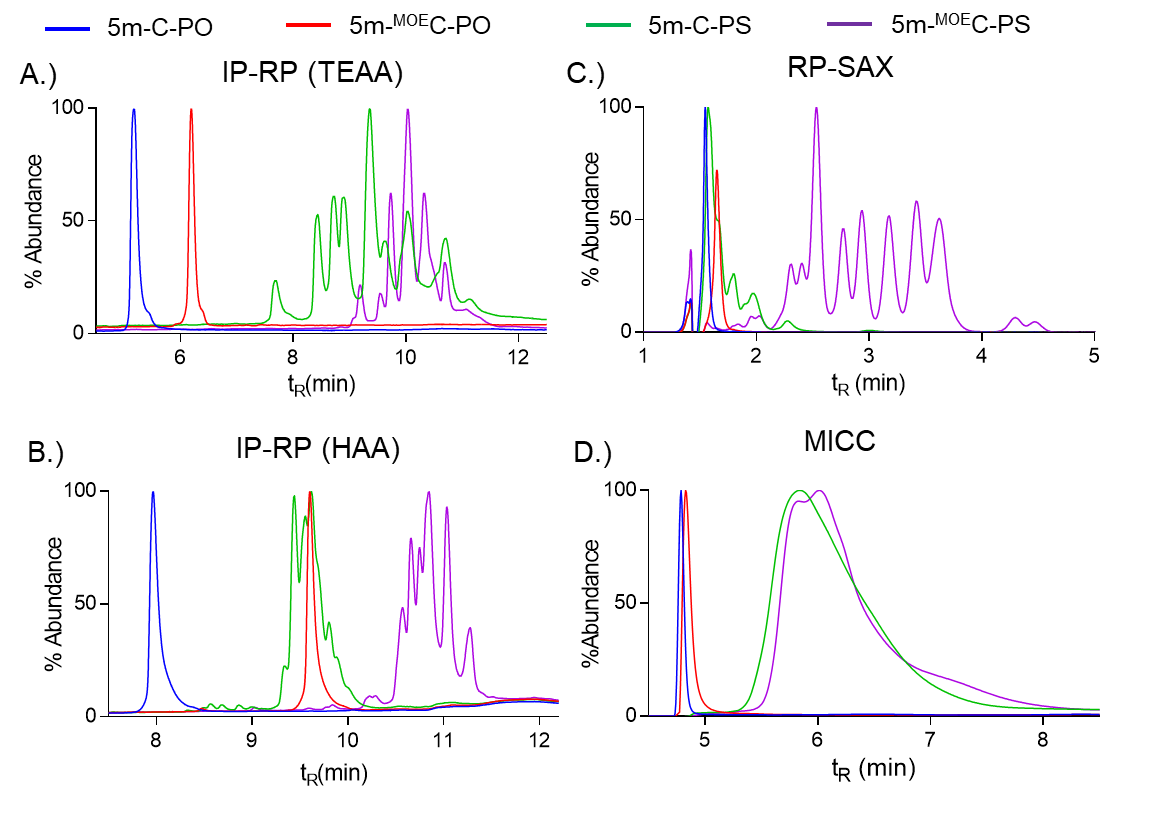


**SI Figure S2**. LC separation of homomeric 5-mers with and without 2'-^MOE^ modification and with PO- or PS-linkages. A.) IR-RP using TEAA, B.) IR-RP using HAA, C.) RP-SAX, and D.) MICC.


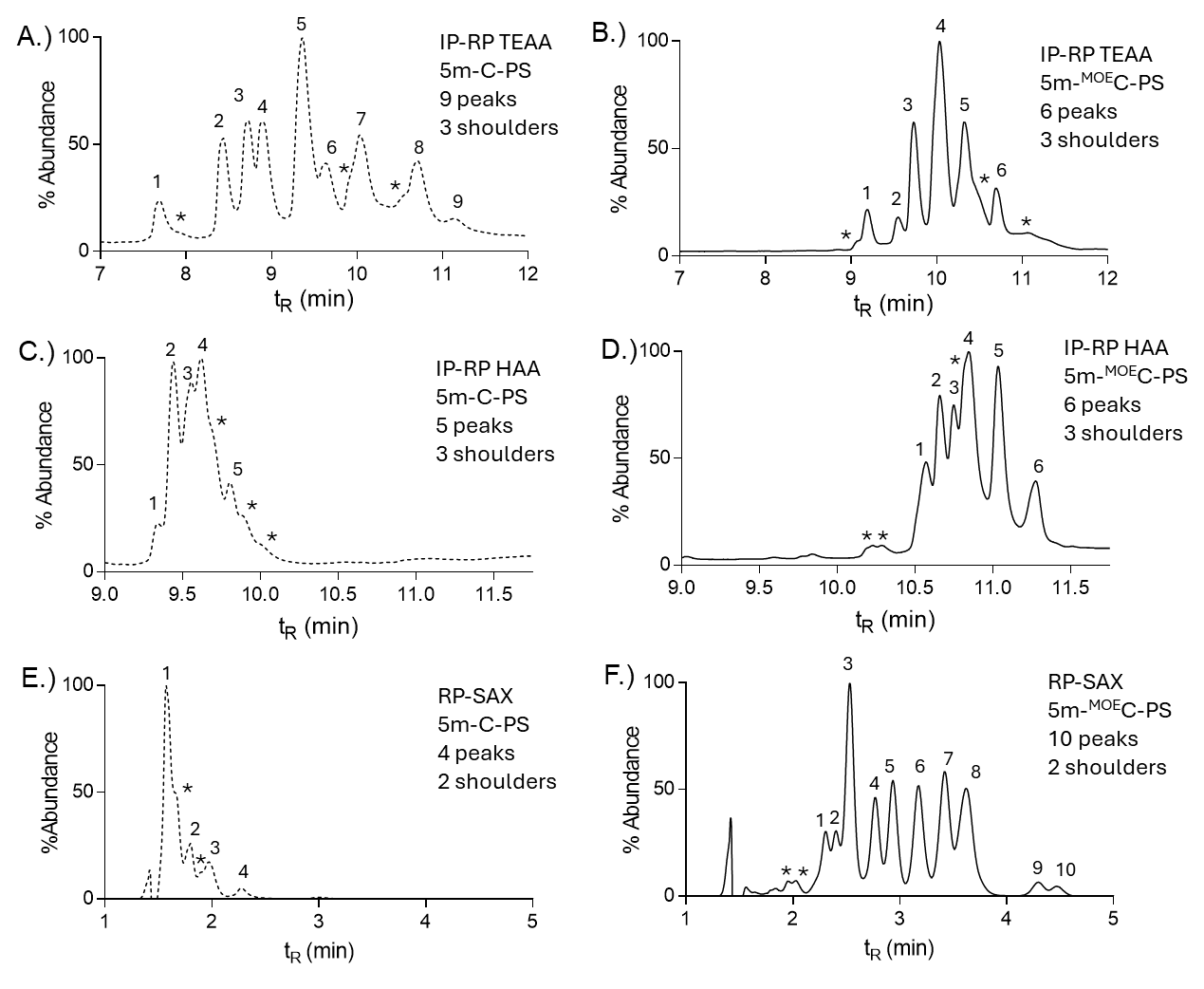


**SI Figure S3**. LC separation of PS-linked homomeric 5-mers with and without 2'-^MOE^ modification. A.) IR-RP using TEAA for 5m-C-PS, B.) IR-RP using TEAA for 5m-C- ^MOE^-PS, C.) IR-RP using HAA for 5m-C-PS, D.) IR-RP using HAA for 5m-C- ^MOE^-PS, E.) RP-SAX for 5m-C-PS, F.) RP-SAX for 5m-C- ^MOE^-PS.


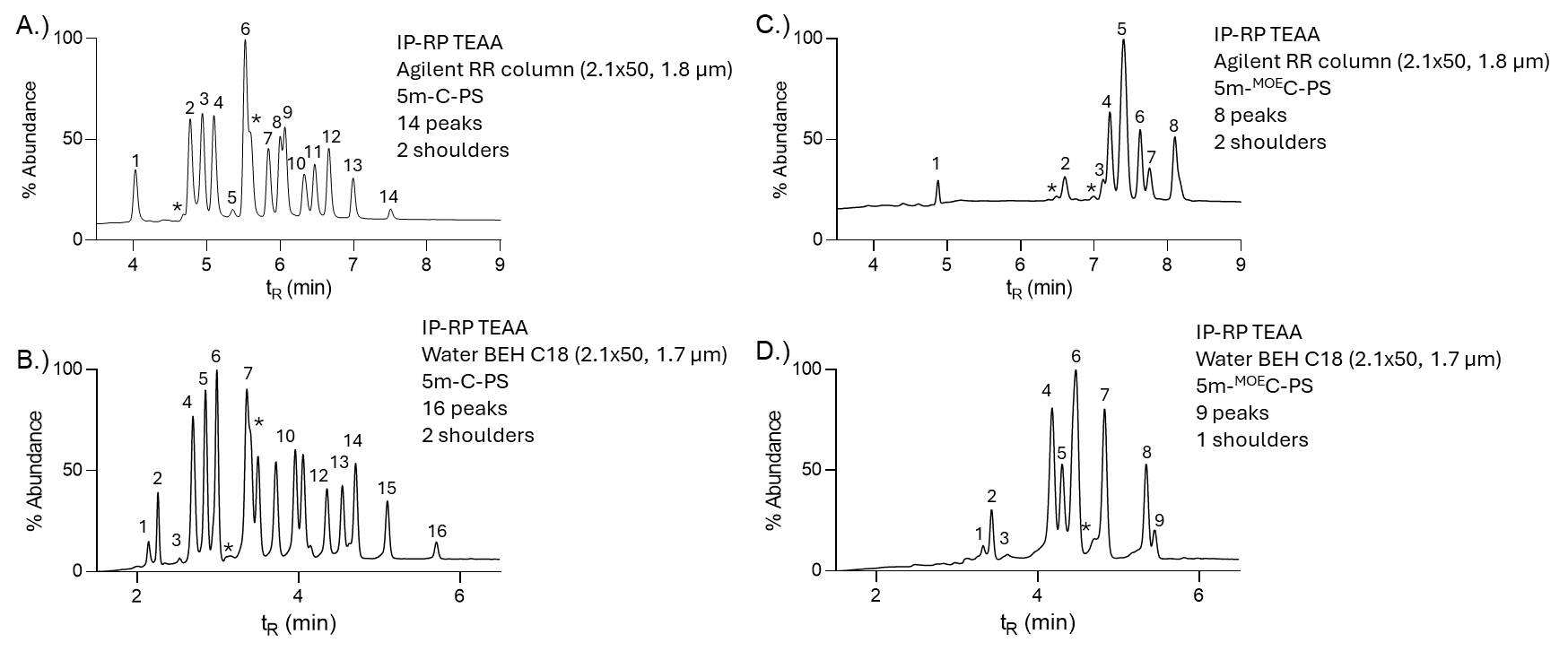


**SI Figure S4.** LC separation of PS-linked homomeric 5-mers with and without 2'-^MOE^ modification. A.) IR-RP using TEAA for 5m-C-PS on an Agilent RR column, B.) IR-RP using TEAA for 5m-C-PS on a Waters BEH C18, C.) IR-RP using TEAA for 5m-^MOE^C-PS on an Agilent RR column, D.) IR-RP using TEAA for 5m-^MOE^C-PS on a Waters BEH C18.


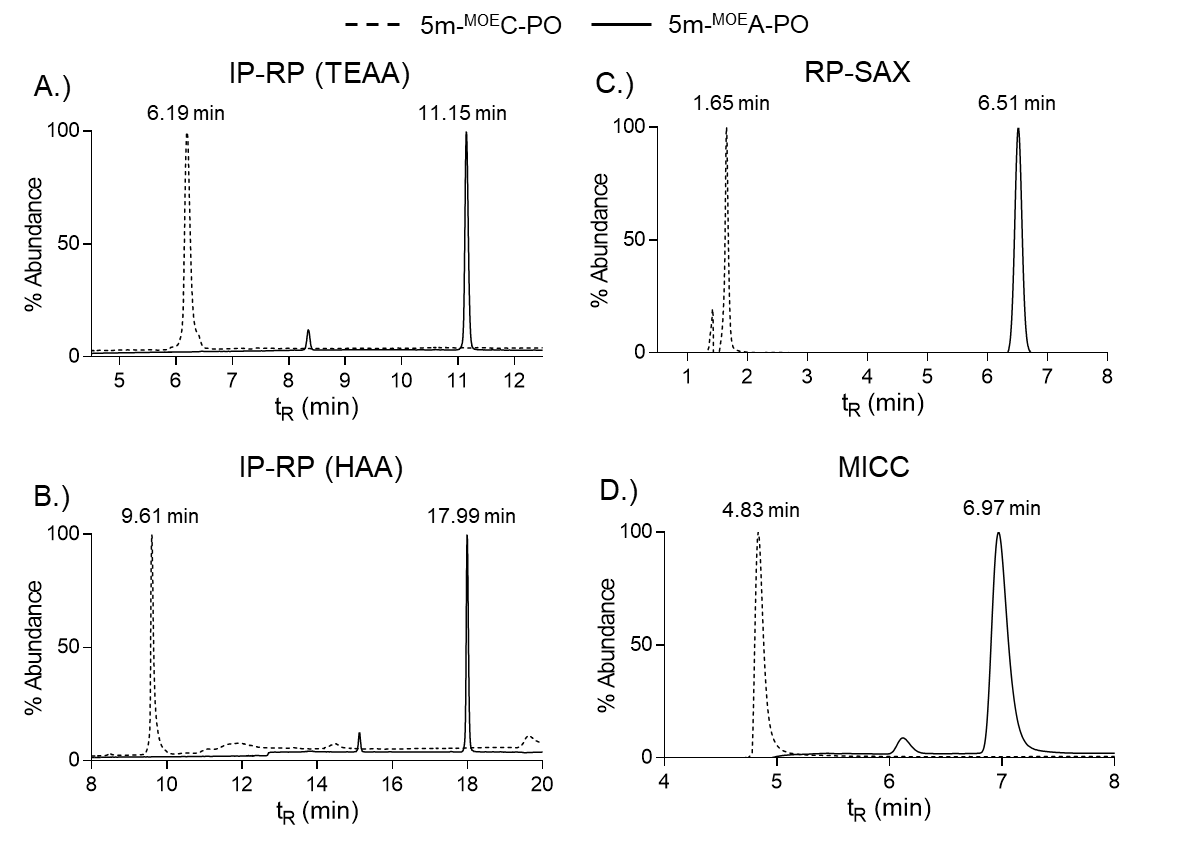


**SI Figure S5.** LC separation of PO-linked homomeric and heteromeric 5-mers with 2'-^MOE^ modification. A.) IR-RP using TEAA, B.) IR-RP using HAA, C.) RP-SAX, D.) MICC.


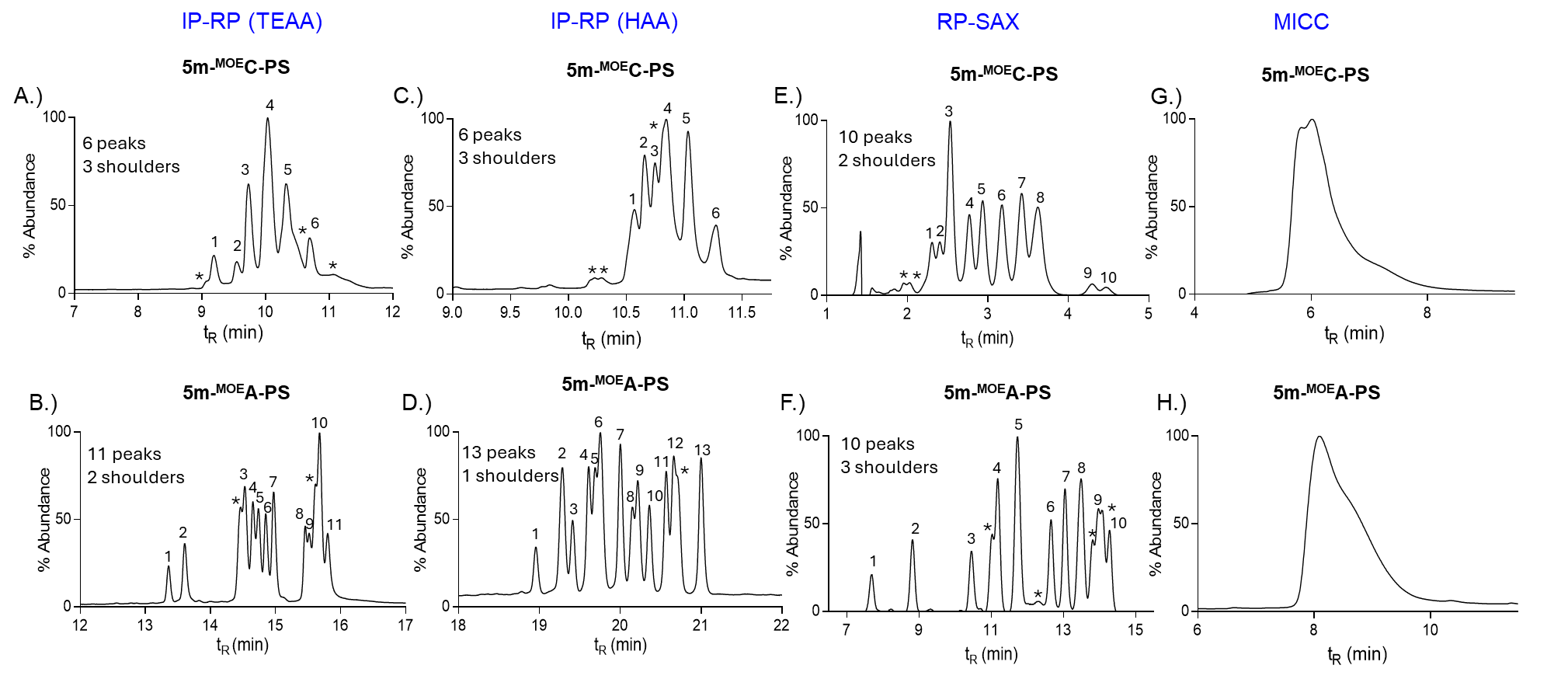


**SI Figure S6.** LC separation of PS-linked homomeric and heteromeric 5-mers with 2'-^MOE^ modification. A.) IR-RP using TEAA for 5m-^MOE^C-PS, B.) IR-RP using TEAA for 5m-^MOE^A-PS, C.) IR-RP using HAA for 5m-^MOE^C-PS, D.) IR-RP using HAA for 5m-^MOE^A-PS, E.) RP-SAX for 5m-^MOE^C-PS, F.) RP-SAX for 5m-^MOE^A-PS, G.) MICC for 5m-^MOE^C-PS, H.) MICC for 5m-^MOE^A-PS.
